# Prognostic value of neutrophil-to-lymphocyte ratio in urothelial carcinoma of the upper urinary tract and bladder: a systematic review and meta-analysis

**DOI:** 10.18632/oncotarget.17467

**Published:** 2016-04-27

**Authors:** Xintao Li, Xin Ma, Lu Tang, Baojun Wang, Luyao Chen, Fan Zhang, Xu Zhang

**Affiliations:** ^1^ Department of Urology, State Key Laboratory of Kidney Disease, Chinese PLA Medical Academy, Chinese People’s Liberation Army General Hospital, Beijing, China

**Keywords:** inflammation, neutrophil-to-lymphocyte ratio, urothelial cancer, prognosis, meta-analysis

## Abstract

The neutrophil-to-lymphocyte ratio (NLR) is an inflammation marker that has prognostic value for various tumors, but its prognostic value in urothelial carcinoma (UC) remains controversial. This meta-analysis investigated the prognostic value of NLR in UC. A systematic search was performed on PubMed, ISI Web of Science, and Embase for studies focusing on the association between NLR and clinical features or prognosis of UC and published until November 2016. Prognostic outcomes and clinical features were collected and analyzed. A total of 11,538 patients from 32 studies were included in the meta-analysis. Increased pretreatment NLR predicted poor overall survival (hazard ratio [HR] = 1.72, 95% confidence interval [CI] = 1.45–2.05), progression free survival (HR = 1.68, 95% CI = 1.44–1.96), and cancer specific survival (HR = 1.64, 95% CI = 1.39–1.93) in all the patients. The increased pretreatment NLR was correlated with increased lymphovascular invasion (HR = 1.29, 95% CI = 1.17–1.43), high tumor T stage (HR = 1.25, 95% CI = 1.12–1.39), and tumor grade (HR = 1.07, 95% CI = 1.01–1.14) but not with lymph node involvement, carcinoma in situ, multifocality, or positive margin. Our meta-analysis indicated that NLR could predict the prognosis for UC and was associated with UC progression in terms of lymphovascular invasion, tumor T stage, and tumor grade.

## INTRODUCTION

Upper tract urothelial carcinoma (UTUC) currently accounts for 5%-10% of all urologic tumors [1-3], whereas bladder cancer (BC) remains the most common malignancy of the urinary tract, the 7th most common cancer in men, and the 17th most common cancer in women [4]. Radical nephroureterectomy (RNU) and radical cystectomy (RC) are the surgical standards for muscle invasive tumors. However, the prognosis of patients undergoing RNU or RC remains poor because of the risk of disease recurrence or metastasis [4-6]. Carcinoma *in situ* (CIS) is a precursor lesion of invasive cancer and associated with an increased risk of disease progression and recurrence [7]. Neuroendocrine tumors in the bladder are rare and malignant, and these tumors are accounted for < 1% of all urinary bladder malignancies. Approximately 60% of patients suffer from metastatic diseases at the time of diagnosis [8].

Inflammation is implicated in cancer development and progression [9, 10] and associated with poor prognosis in some cancers. Inflammation may also severely affect tumor microenvironments to facilitate cancer progression [11]. Thus, systematic inflammation markers may serve as prognostic biomarkers [12, 13]. Neutrophil-to-lymphocyte ratio (NLR) is also an indicator of general immune responses to various stress stimuli and is correlated with the prognosis of various tumor types [14-17].

The prognostic significance of pretreatment NLR in patients with UTUC or BC has been evaluated, and results have shown that NLR can be used as a prognostic marker for UC [18-23]. However, contradicting conclusions have been presented in other studies [24, 25], and related results remain controversial. In a previous meta-analysis on the NLR of patients with UTUC and BC, NLR can predict cancer-specific survival (CSS), progression-free survival (PFS), and overall survival (OS). Nevertheless, this meta-analysis included 11 original studies and did not report the correlation between NLR and clinical features. Although NLR and UC have been extensively investigated, the relationship between NLR and urethral cancer has yet to be described. Thus, we performed a systematic review and meta-analysis on published studies to determine the predictive value of NLR for the clinical features and prognosis of UTUC and BC.

## RESULTS

### Search results

**Figure 1:**
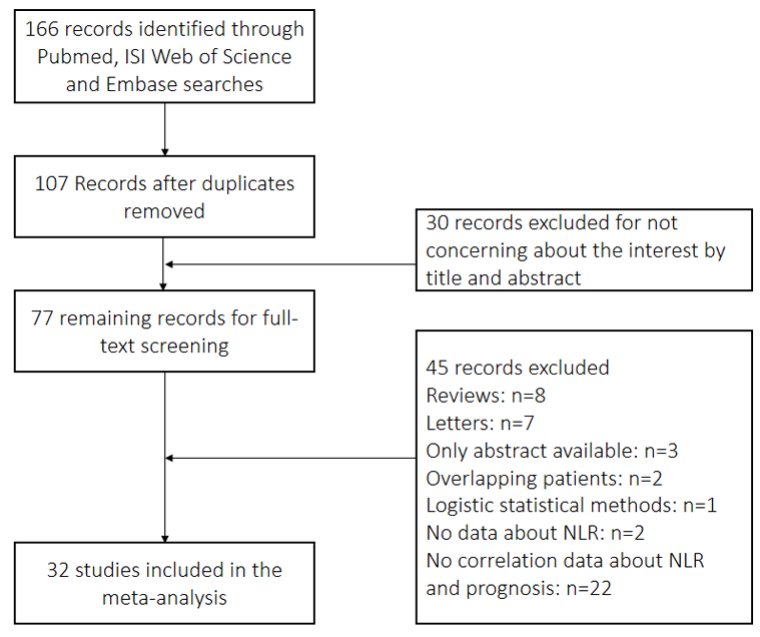
Flowchart of selection of studies for inclusion in meta-analysis

A flow chart of the selection of literature is shown in Figure 1. A total of 166 records were retrieved using the search strategy, and 107 of these records were obtained after duplicated studies were excluded. Of the 107 records, 30 irrelevant studies were excluded after their titles and abstracts were initially evaluated. The full texts of the 77 remaining articles were then assessed. A total of 45 records were excluded: 8 reviews, 7 letters, 3 abstracts, 2 overlapping cases, 1 logistic statistical method, 2 unrelated to NLR, and 22 without correlation between NLR and prognosis or clinical features. The three abstracts didn’t provided enough information for analysis. The manuscript using logistic statistical method didn’t include follow-up information. The two unrelated studies were focused on lymphocyte to monocyte ratio and derived NLR. The full texts of the 32 remaining articles were assessed.

Overall, 32 studies with 11,538 patients were included in the meta-analysis [18-49]. These studies evaluated the prognostic value of pretreatment NLR in UC and were reported within 5 years.

### Characteristics of eligible studies

The PRISMA checklist is shown in Supplemental Table 1. The detailed information on the 32 studies is listed in Supplemental Tables 2, 3, and 4. These studies included 9 from Japan, 4 from China, 4 from Korea, 3 from US, 2 from Australia, 2 from Canada, 2 from Turkey, 1 from Israel, 2 from Italy, 1 from England, 1 from Spain, and 1 from the European multicenter data. These 11,538 patients in these studies had a median number of 214 patients (range 23-2477; mean:360.6±456.9) per study. All of the studies were retrospective observational cohort studies. Of the 32 studies, 19 included non-muscle-invasive or muscle-invasive BC patients, 11 included patients with UTUC, and 2 included UC patients. All NLRs were detected before treatment was administered. The following studies included patients who underwent specific procedures: 13 studies, RC; 10 studies, RNU; 6 studies, transurethral resection of bladder tumor; and 4 studies, chemotherapy with cisplatin or pemetrexed. No other therapy were provided in the original research. The median follow-up time was 40 months (range of 11.5-130.8 months). The relationship between NLR and OS was investigated in 17 cohorts (9 by multivariate analysis). The relationship between NLR and PFS were explored in 19 cohorts (13 by multivariate analysis). Lastly, the relationship between NLR and CSS were examined in 17 cohorts (12 by multivariate analysis). Furthermore, the associations between NLR and several clinical features, such as lymph node involvement (LNI), lymphovascular invasion (LVI), tumor T stage, tumor grade, CIS, multifocality, and positive margin, were investigated in 8, 9, 10, 11, 12, 9, and 6 studies, respectively.

### Effect of NLR on OS in patients with UC

**Figure 2:**
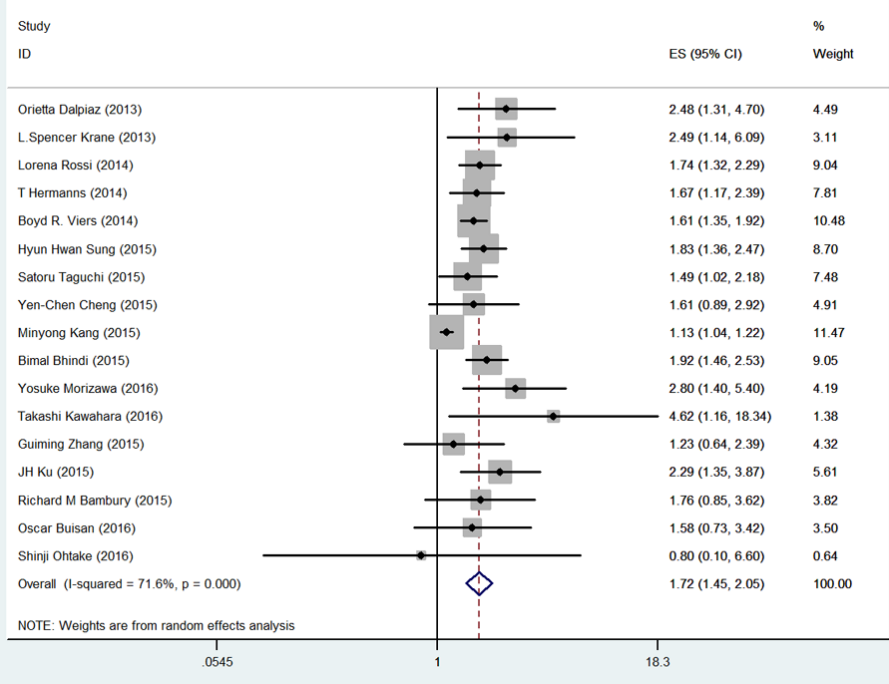
Meta-analysis of NLR value and OS in UC patients

The association between NLR and OS was evaluated in 17 cohorts that included 4,432 patients. The OS of the patients with high pretreatment NLR was significantly poorer than that of the patients with low NLR (HR = 1.72, 95% CI = 1.45-2.05; Figure 2 and Table 1). Significant heterogeneity among these studies was observed (I^2^ = 71.6%, *p* < 0.001). Thus, pooled analyses were performed using a random-effect model.

**Table 1:** Results of the meta-analysis on predictive value of NLR in UC

	OS				PFS				CSS			
	*n*	HR	LCI	UCI	*n*	HR	LCI	UCI	*n*	HR	LCI	UCI
**Overall**	17	1.72	1.45	2.05	19	1.68	1.44	1.96	17	1.64	1.4	1.93
**Geographic area**												
**1.Asian**	9	1.72	1.45	2.19	12	1.8	1.51	2.15	11	1.74	1.36	2.21
**2.non-Asian**	8	1.73	1.54	1.95	7	1.49	1.2	1.85	6	1.56	1.23	1.99
**statistical methods**												
**1.univariate**	15	1.834	1.672	2.012	15	1.81	1.55	2.11	13	1.95	1.62	2.34
**2.multivariate**	9	1.91	1.43	2.57	13	1.77	1.42	2.2	12	1.66	1.35	2.04
**Patient**												
**1.Localized**	12	1.78	1.59	1.99	17	1.73	1.45	2.07	15	1.74	1.46	2.07
**2.metastatic**	4	1.65	1.33	2.03	2	1.52	1.18	1.96	1	1.48	1.01	2.17
**sample size**												
**1.<230**	10	1.78	1.44	2.2	8	2.36	1.71	3.27	7	1.94	1.54	2.43
**2.>=230**	7	1.65	1.32	2.08	11	1.54	1.31	1.81	10	1.5	1.26	1.79
**NLR standard**												
**1.<2.65**	8	1.72	1.25	2.37	9	2	1.63	2.47	7	1.92	1.35	2.74
**2.>2.65**	9	1.72	1.54	1.92	10	1.48	1.24	1.77	10	1.52	1.27	1.82
**follow-up**												
**1.=<39**	8	1.68	1.23	2.31	6	1.62	1.29	2.04	8	1.58	1.25	1.99
**2.>39**	7	1.72	1.54	1.92	11	1.66	1.36	2.03	8	1.77	1.37	2.28
**tumor type**												
**1.UTUC**	5	1.75	1.48	2.06	8	1.53	1.21	1.94	9	1.67	1.34	2.09
**2.BC**	11	1.71	1.35	2.16	11	1.8	1.54	2.11	8	1.65	1.26	2.18

NLR, Neutrophil-to-lymphocyte ratio; OS, overall survival; PFS, progression free survival; CSS, cancer specific survival; BC, bladder cancer; UTUC, upper tract urothelial carcinoma; HR, hazard ratio; LCI, lower confidence interval; UCI, upper confidence interval.

### Effect of NLR on PFS in patients with UC

**Figure 3:**
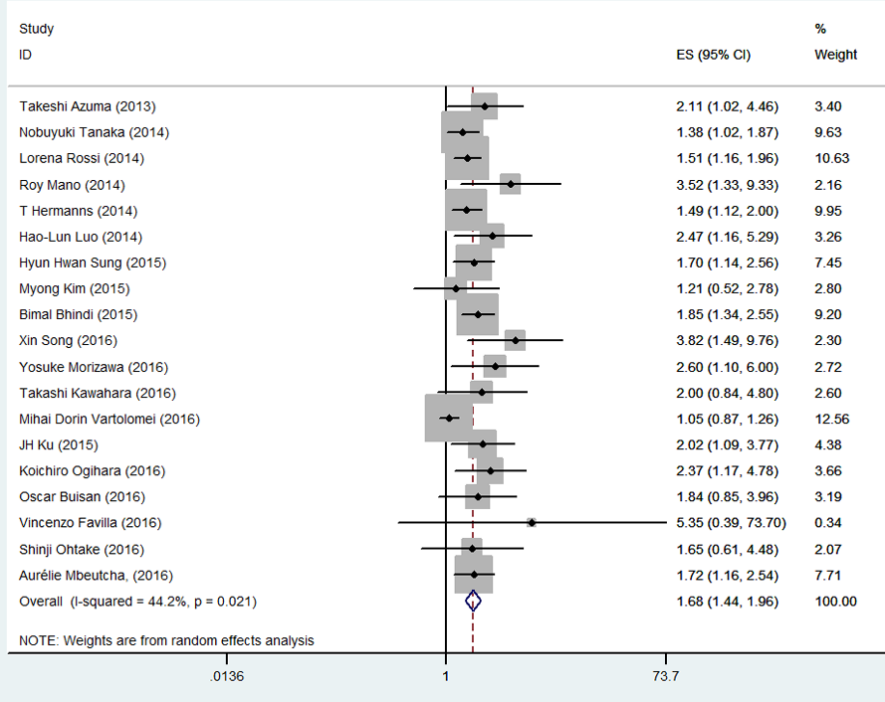
Meta-analysis of NLR value and PFS in UC patients

Of the included studies, 19 investigated the association between NLR and PFS. Among the 8,182 patients in these studies, the patients with high pretreatment NLR exhibited poorer PFS than those with low NLR did (HR = 1.68, 95% CI 1.44-1.96; Figure 3 and Table 1). Significant heterogeneity among these studies was found (I^2^ = 44.2%, *p* = 0.021), and these data were analyzed using a random-effect model.

### Effect of NLR on CSS in patients with UC

**Figure 4:**
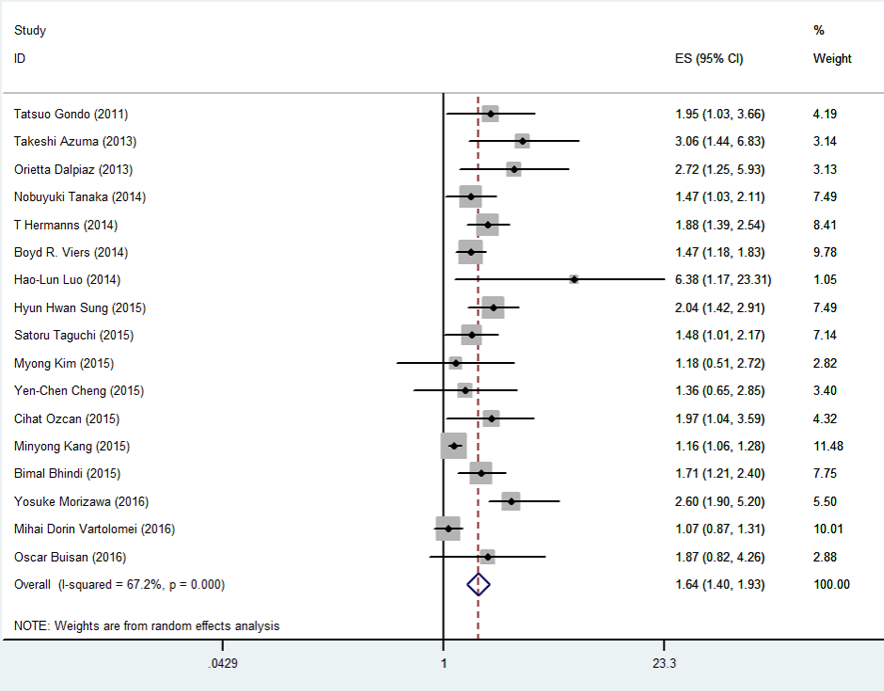
Meta-analysis of NLR value and CSS in UC patients

The association between NLR and CSS was investigated in 17 studies involving 7645 patients. Similarly, the patients with high pretreatment NLR had a significantly poorer CSS than those with low NLR (HR = 1.64, 95% CI = 1.39-1.93; Figure 4 and Table 1). Significant heterogeneity among these studies was also found (I^2^ = 67.2%, *p* < 0.001), and pooled analysis was conducted using a random-effect model.

We explored the heterogeneity among the studies by performing subgroup and meta-regression analyses (Supplemental Table 5). In the subgroup analysis of OS, a higher pretreatment NLR was associated with poorer OS in all of the subgroup analyses in terms of geographic area, statistical methods, tumor metastatic status, sample size, NLR standard, follow-up time, and tumor type (Table 1). The same results were achieved in the subgroup analyses of PFS and CSS.

Meta-regression analysis suggested that tumor type (*p* = 0.011) could partially account for the source of the heterogeneity of the pooled PFS and tumor type (*p* = 0.013). Geographic area (*p* = 0.032) and follow-up time (*p* = 0.045) could partially explain the source of the heterogeneity of the pooled CSS analysis results.

### Correlation between NLR and clinicopathological features

The correlations between NLR and clinical features of UC are presented in Table 2, and 8, 9, 10, 11, 12, 9, and 6 studies were available for the pooled analysis of LNI, LVI, tumor T stage, tumor grade, CIS, multifocality, and positive margin, respectively. NLR was positively correlated with LVI (HR = 1.29, 95% CI = 1.17-1.43), high tumor T stage (HR = 1.25, 95% CI = 1.12-1.39), and tumor grade (HR = 1.07, 95% CI = 1.01-1.14) but not with LNI, CIS, multifocality, and positive margin (Supplemental Figures 1-7).

**Table 2:** The meta-analysis of NLR and clinical features

	n	HR	LCI	UCI	Heterogeneity		Publication bias	
					*P*a	I2(%)	Pc(Begg's test)	Pd(Egger'test)
**LNI**	8	1.358	0.93	1.983	0.006	64.8	1	0.742
**LVI**	9	1.29	1.17	1.43	0.426	0.9	0.602	0.707
**CIS**	10	1.05	0.85	1.3	0	80.1	0.721	0.917
**T stage**	11	1.25	1.12	1.39	0	69.6	1	0.331
**tumor grade**	12	1.07	1.01	1.14	0.021	50.9	0.086	0.065
**multifocality**	9	1.04	0.87	1.25	0.001	70.5	0.251	0.317
**positive margin**	6	1.45	0.96	2.19	0.135	40.6	1	0.961

NLR, Neutrophil-to-lymphocyte ratio; HR, hazard ratio; LCI, lower confidence interval; UCI, upper confidence interval; LNI, lymphnode involvement; LVI, lymphovascular invasion; CIS, carcinoma *in situ*

The combined OR for LNI, CIS, and T stage was calculated using the random-effect model because of the heterogeneity among the studies. LVI, tumor grade, multifocality, and positive margin was subjected to pooled analysis by using the fixed-effect model.

### Publication bias analysis

**Figure 5:**
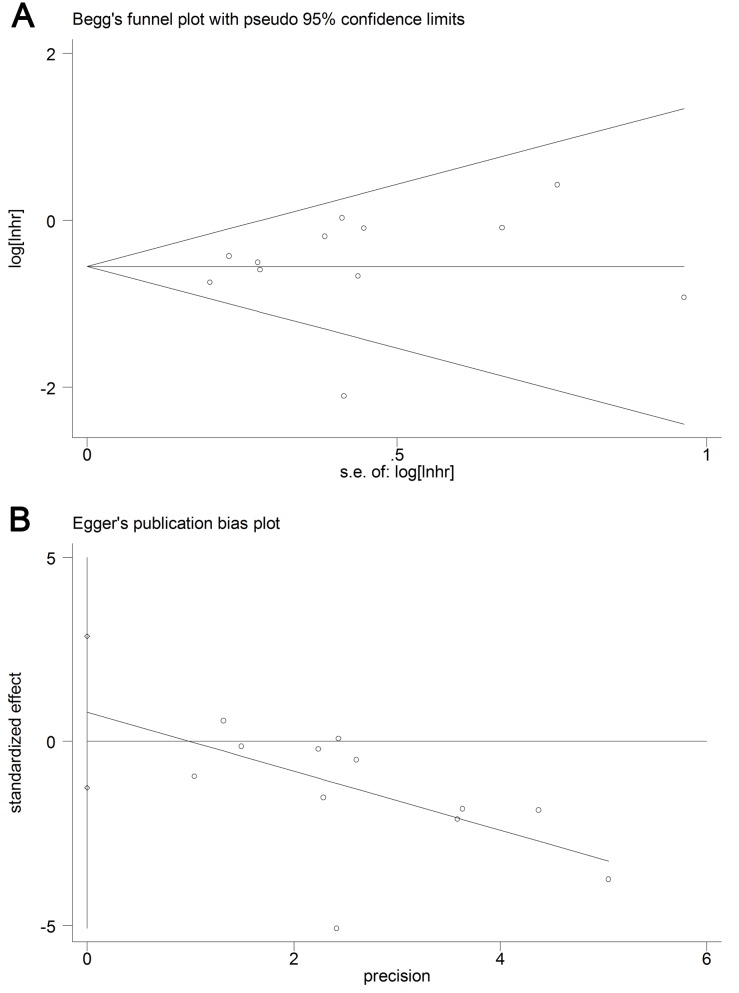
Begg’s and Egger’s test results for UC patients’ OS

The funnel plots for OS, PFS and CSS were symmetrical. The p-values of Begg’s and Egger’s tests indicated the presence of publication bias in terms of OS (*p* = 0.329 and *p* = 0.414), PFS (*p* = 0.502 and *p* = 0.597), and CSS (*p* = 0.583 and *p* = 0.908) among the included studies (Figure 5, Supplemental Figures 8 and 9).

### Sensitivity analysis

A sensitivity analysis was performed by deleting one study at a time (Supplemental Figures 10-12). The changes in the pooled HRs for OS, PFS, and CSS were insignificant. Thus, our results were reliable.

## DISCUSSION

Inflammation is a critical step in the pathogenesis and progression of cancer [9, 10]. The excess production of pro-inflammatory cytokines commonly results in an acute phase reaction in cancer patients [50]. In addition, adipose inflammation and epithelial-mesenchymal transition are involved in inflammation induced tumor initiation and progression [51, 52]. Tumors can also harness the inflammatory responses *via* various inflammatory mediators to survive and proliferate, and even fostering a tumor milieu ripe for progression and metastasis [53]. Now more evidences show that the presence of systemic inflammation is correlated with poorer survival in several cancers [54-57].

In the present meta-analysis, the results indicated that NLR not only yielded prognostic values for OS, PFS, and CSS in patients with UC but also predicted the presence of LVI, high tumor T stage, and tumor grade. With the advantage of wide availability and low cost, NLR can be a potential marker for patient prognosis and cancer progression.

Subgroup analysis was performed because of the significance of heterogeneity among the included studies. Our results showed that higher NLR predicted poor survival in each subgroup in terms of OS, PFS, and CSS. However, the cut-off NLR has yet to be defined. Published studies used cut-off values from 2 to 5. Our analysis showed that the prediction was significant either in the standard of < 2.65 or > 2.65 subgroup. The subgroup analysis of geographic area suggested that the NLR prediction value was not affected by racial differences. Multivariate subgroup analysis implied that increased NLR independently predicted poor prognosis. The NLR predictive significance did not significantly differ between UTUC and BC. An abstract without a full text showed that NLR is an independent predictor of metastasis-free survival for nonmetastatic UTUC (2.667; 1.069-6.655; *p* = 0.0355) [58]. Albayrak S et al. [59] found that NLR couldn’t predict tumor progression and recurrence of nonmuscle invasive BC after age is corrected. However, localized and metastatic UC did not differ in our meta-analysis.

Our results showed that the high pretreatment NLR increased the presence of LVI, tumor T stage, and tumor grade. Another study concluded that higher preoperative NLR can predict patients who may be upstaged at the time of surgery [60]. Preoperative NLR may be predictive of the pathologic stage in patients with BC larger than 3 cm [61]. These results suggested that NLR may be an important factor that affected UC progression and can serve as a marker for UC progression. In other cancers, NLR is also associated with tumor invasion, lymph node metastasis, vascular invasion [14, 15, 62].

Derived NLR (neutrophil count/white cell count-neutrophil count) can also predict patient prognosis. Van Kessel et al. and Kim Myong et al. [23, 63] showed that higher derived NLR indicated poor prognosis for UTUC and muscle-invasive BC. Postoperative NLR may also be significant in predicting the prognosis. Minyong Kang et al. [29] reported that higher postoperative NLR increased the possibility of progression and prognosis in UC patients. Increased pre- and postoperative NLRs are associated with poor oncologic outcomes. Early postoperative high NLR is also related to increased all-cause postoperative mortality. Another study on advanced UC concluded that pretherapy NLR, follow-up NLR, and change in NLR are correlated with clinical outcomes. However, pretherapy NLR cannot predict objective responses [40]. A study with a limited sample on chemotherapy has shown that changes in NLR during neoadjuvant chemotherapy are associated with pathological responses [64]. These studies showed that postoperative NLR and NLR dynamics are also valuable markers for prognosis.

Heterogeneity was observed among the included studies. Thus, most of the pooled analyses were performed using a random-effect model. Patient pretreatment characteristics, sample size, tumor type, tumor stage, cut-off NLR, and treatment were variable in the included studies. Subgroup analysis revealed that the conclusion was unaffected by these factors. Meta-regression indicated that tumor type, geographic area, and follow-up time can account for some heterogeneity. However, sensitivity analysis demonstrated that our results were reliable.

The present meta-analysis is characterized by some limitations. First, most of the included studies were retrospective and could generate bias during data selection and analysis. Second, the cut-off NLR varied in the included studies from 2 to 5, which resulted in heterogeneity in the analysis. Third, the Newcastle-Ottawa Quality Assessment Scale (NOS) score obtained in several studies was lower than that in other studies. Some of these studies didn’t compare the baseline characteristics while other studies did not provide accurate follow-up durations. Hence, these factors might influence the final results. Fourth, NLR might be affected by other causes, such as valvular heart diseases, acute coronary syndromes, liver diseases, renal diseases, hypertension, inflammatory diseases, infection, other tumor types, and medications. These factors were unlikely included in the pooled analysis.

Despite these limitations, our study supported the prognostic significance of NLR in UC. With the convenience and cost-effectiveness of available NLR, this parameter can be used for the prognostic prediction and surveillance of cancer progression.

## CONCLUSIONS

NLR is a useful predictive marker for the progression of UC and prognosis of patients with UC. Patients with high pretreatment NLR exhibit poor OS, PFS, and CSS. High pretreatment NLR increases the presence of lymphovascular invasion, tumor T stage and tumor grade. These prognostic values are significant for UTUC, BC and metastatic UC. In addition, higher NLR also predicts postoperative metastasis. Postoperative NLR and derived NLR are both suggested promising prognostic markers for UC. Further studies assessing other inflammation markers in combination with NLR are required to evaluate their prognostic values in UC.

## MATERIALS AND METHODS

### Search strategy

A systematic literature search was performed on PubMed, ISI Web of Science, and Embase until November 2016. Search terms included “NLR,” “neutrophil to lymphocyte ratio,” “neutrophil-lymphocyte ratio,” “bladder,” ”urothelial,” and “tumor, cancer, neoplasm, or carcinoma.” First, duplicated studies were excluded. Second, titles and abstracts were scanned thoroughly to exclude irrelevant articles. Finally, all of the full texts of the remaining articles were assessed comprehensively to identify the studies that contained the topic of interest.

### Selection criteria

The studies included in the meta-analysis were randomized controlled studies or observational studies (case-control or cohort) that evaluated the association between pretreatment NLR and UC prognosis. The studies were included if they (1) included patients histopathologically diagnosed with UC, (2) provided pretreatment NLR and reported cut-off values, (3) focused on the prognosis or clinical features of UC, and (4) analyzed the associations between pretreatment NLR and survival outcomes or clinical features (OS, PFS, CSS, LNI, LVI, tumor T stage, tumor grade, CIS, multifocality and positive margin). The full texts of the studies must be available to obtain these data. The exclusion criteria were (1) non-English papers; (2) review articles, editorial comments, letters, expert opinion, conference abstracts, or case reports; (3) overlapping or duplicate data; (4) animal models or cancer cells; and (5) insufficient data for estimating HRs and 95% CIs.

All of the evaluations were conducted independently by two reviewers (X.M and L.T) to ensure the accurate inclusion of the studies. Both reviewers are urologists proficient in urology, epidemiology, and statistics. When several studies contained overlapping data, the study with the largest data was included. Multivariate outcomes were preferred over univariate outcomes if both were provided in the original study. Univariate outcomes were acceptable if no multivariate results were presented. The survival or mortality curves provided in the original study were used to calculate the values. If the manuscripts contained insufficient data, the supplemental data in these manuscripts were searched. If the data were still insufficient, we contacted the authors for additional essential data.

### Data extraction

Data were extracted by two independent reviewers (X.M and L.T). Disagreements in data extraction were resolved through a consensus. The NOS was used to assess the qualities of the included studies. The evaluation included three aspects, namely selection, comparability, and outcomes in the case and control groups. Studies with scores of ≥6 were defined as high-quality studies. The following relevant data were extracted in a predefined table: author, year, country, tumor type, age, patient sample, follow-up duration, cut-off score, NLR (high/low), treatment, and endpoint (OS, PFS, CSS, LNI, LVI, tumor T stage, tumor grade, CIS, multifocality, and positive margin).

Some studies presented survival data using Kaplan-Meier curves. Therefore we used GetData Graph Digitizer 2.26 (http://getdata-graph-digitizer.com/, Russia) to digitize and extract the relevant survival data. We calculated HRs by using the extracted data from the original studies through the methods reported by Tierney et al. [65].

The cut-off values for NLR varied among the studies. Thus, we defined the NLR standard according to the standards used in the original study.

### Statistical analysis

This meta-analysis was performed using Stata version 12.0 (StataCorp LP, TX, USA), and statistical analysis was conducted according to the guidelines proposed by the Meta-analysis of Observational Studies in Epidemiology group. The associations between NLR and outcomes were reported as HRs and 95% CIs, either obtained directly from the original studies or calculated from indirect data. For the analysis of the relationship between NLR and clinical features, ORs and 95% CIs were considered effective values. Heterogeneity among the studies was determined through Q and I^2^ tests. A fixed-effect model was performed in the absence of significant heterogeneity. Otherwise, a random-effect model was used. Potential publication bias was identified by Begg’s and Egger’s tests. The influence of publication bias on the overall effect was assessed by the “trim and fill” method described by Duval et al. [66]. A *p*-value of < 0.05 was considered statistically significant. All p-values were two tailed.

Subgroup analyses were performed to investigate the associations of NLR with clinical features and NLR and prognosis in relation to geographic area, statistical methods, tumor type (localized or metastatic status), sample size, NLR cut-off value ( < 2.65 and ≥2.65), follow-up duration, and tumor type (UTUC or BC). Meta-regression was then performed to investigate the source of heterogeneity. Pooled analysis was performed on the association between NLR value and clinical features because of the limited studies. Furthermore, sensitivity analysis was performed to examine the robustness of the pooled results. The meta-analysis followed the standard PRISMA checklist.

## SUPPLEMENTARY MATERIALS FIGURES AND TABLES


