# Supplementary material for: Prognostic value of neutrophil-to-lymphocyte ratio in urothelial carcinoma of the upper urinary tract and bladder: a systematic review and meta-analysis
Source: Oncotarget. 2016 Apr 27;8(37):62681–92. doi: 10.18632/oncotarget.17467 (PMC5617540; doi:10.18632/oncotarget.17467)
Supplement: Supplementary file 1 [file oncotarget-08-62681-s001.pdf]

# Prognostic value of neutrophil-to-lymphocyte ratio in urothelial carcinoma of the upper urinary tract and bladder: a systematic review and meta-analysis

**Supplemental data**

**Supplementary Table 1 - Checklist of items to include when reporting a systematic review or meta-analysis**

| Section/topic | # | Checklist item | Reported on page # |
|---|---|---|---|
| **TITLE** | | | |
| Title | 1 | Identify the report as a systematic review, meta-analysis, or both. | Page 1 |
| **ABSTRACT** | | | |
| Structured summary | 2 | Provide a structured summary including, as applicable: background; objectives; data sources; study eligibility criteria, participants, and interventions; study appraisal and synthesis methods; results; limitations; conclusions and implications of key findings; systematic review registration number. | Page 2 |
| **INTRODUCTION** | | | |
| Rationale | 3 | Describe the rationale for the review in the context of what is already known. | Page 3,4 |
| Objectives | 4 | Provide an explicit statement of questions being addressed with reference to participants, interventions, comparisons, outcomes, and study design (PICOS). | Page 3,4 |
| **METHODS** | | | |
| Protocol and registration | 5 | Indicate if a review protocol exists, if and where it can be accessed (e.g., Web address), and, if available, provide registration information including registration number. | |
| Eligibility criteria | 6 | Specify study characteristics (e.g., PICOS, length of follow-up) and report characteristics (e.g., years considered, language, publication status) used as criteria for eligibility, giving rationale. | Page 12,13 |
| Information sources | 7 | Describe all information sources (e.g., databases with dates of coverage, contact with study authors to identify additional studies) in the search and date last searched. | Page 12 |
| Search | 8 | Present full electronic search strategy for at least one database, including any limits used, such that it could be repeated. | Page 12 |

| | | | |
|---|---|---|---|
| Study selection | 9 | State the process for selecting studies (i.e., screening, eligibility, included in systematic review, and, if applicable, included in the meta-analysis). | Page 12,13 |
| Data collection process | 10 | Describe method of data extraction from reports (e.g., piloted forms, independently, in duplicate) and any processes for obtaining and confirming data from investigators. | Page 12,13 |
| Data items | 11 | List and define all variables for which data were sought (e.g., PICOS, funding sources) and any assumptions and simplifications made. | Page 13,14 |
| Risk of bias in individual studies | 12 | Describe methods used for assessing risk of bias of individual studies (including specification of whether this was done at the study or outcome level), and how this information is to be used in any data synthesis. | Page 13,14 |
| Summary measures | 13 | State the principal summary measures (e.g., risk ratio, difference in means). | Page 13,14 |
| Synthesis of results | 14 | Describe the methods of handling data and combining results of studies, if done, including measures of consistency (e.g., $I^2$) for each meta-analysis. | Page 13,14 |

| Section/topic | # | Checklist item | Reported on page # |
|---|---|---|---|
| Risk of bias across studies | 15 | Specify any assessment of risk of bias that may affect the cumulative evidence (e.g., publication bias, selective reporting within studies). | Page 13,14 |
| Additional analyses | 16 | Describe methods of additional analyses (e.g., sensitivity or subgroup analyses, meta-regression), if done, indicating which were pre-specified. | Page 13,14 |
| **RESULTS** | | | |
| Study selection | 17 | Give numbers of studies screened, assessed for eligibility, and included in the review, with reasons for exclusions at each stage, ideally with a flow diagram. | Page 4, Figure 1 |
| Study characteristics | 18 | For each study, present characteristics for which data were extracted (e.g., study size, PICOS, follow-up period) and provide the citations. | Page 5,Table S2,3,4 |
| Risk of bias within studies | 19 | Present data on risk of bias of each study and, if available, any outcome level assessment (see item 12). | Page7, Table S5,Table 2 |
| Results of individual studies | 20 | For all outcomes considered (benefits or harms), present, for each study: (a) simple summary data for each intervention group (b) effect estimates and confidence intervals, ideally with a forest plot. | Table S3,4 |
| Synthesis of results | 21 | Present results of each meta-analysis done, including confidence intervals and measures of consistency. | Page 6,7 Figure 2-4,Figure S1-7, Table 1,2 |
| Risk of bias across studies | 22 | Present results of any assessment of risk of bias across studies (see Item 15). | Page7, Figure 5, Figure S8,9 |
| Additional analysis | 23 | Give results of additional analyses, if done (e.g., sensitivity or subgroup analyses, meta-regression [see Item | Page 7,8, |

| | | | |
|---|---|---|---|
| | | 16]). | Figure S10-12 |
| **DISCUSSION** | | | |
| Summary of evidence | 24 | Summarize the main findings including the strength of evidence for each main outcome; consider their relevance to key groups (e.g., healthcare providers, users, and policy makers). | Page 8,9,10 |
| Limitations | 25 | Discuss limitations at study and outcome level (e.g., risk of bias), and at review-level (e.g., incomplete retrieval of identified research, reporting bias). | Page 10,11 |
| Conclusions | 26 | Provide a general interpretation of the results in the context of other evidence, and implications for future research. | Page 14 |
| **FUNDING** | | | |
| Funding | 27 | Describe sources of funding for the systematic review and other support (e.g., supply of data); role of funders for the systematic review. | Page 15 |

*From:* Moher D, Liberati A, Tetzlaff J, Altman DG, The PRISMA Group (2009). Preferred Reporting Items for Systematic Reviews and Meta-Analyses: The PRISMA Statement. PLoS Med 6(7): e1000097. doi:10.1371/journal.pmed1000097

## Supplementary Table 2 Characteristics of included studies

| Study | country | year | tumor | sample | age | Treatment | Cut-off | NLR(high/low) | Detecting time | follow-up(median) | NOS |
|---|---|---|---|---|---|---|---|---|---|---|---|
| **Tatsuo Gondo[1]** | Japan | 2011 | BC | 189 | 68.4（38-85） | RC | 2.5 | 85/104 | Before RC | 25.1(2.1-127.9) | 7 |
| **Takeshi Azuma[2]** | Japan | 2013 | UTUC | 137 | 69.4(40-88) | RNU | 2.5 | 54/83 | 2 weeks before RNU | 60.9(1.9-187.3) | 7 |
| **Orietta Dalpiaz[3]** | Austria | 2013 | UTUC | 202 | 69.3(32-85) | RNU | 2.7 | 128/54 | Before RNU | 45(0-199) | 7 |
| **Nobuyuki Tanaka[4]** | Japan | 2014 | UTUC | 665 | 70(62-76) | RNU | 3 | 184/481 | Before any manipulations | 28(IQR:14-57) | 8 |
| **Mehmet Kaynar[5]** | Turkey | 2014 | BC | 291 | NMIBC:64(27-97); MIBC:70(27-95) | TURBT | 2.5 | 75/216 | Before TURBT | NA | 5 |
| **Lorena Rossi[6]** | Italy | 2014 | UTUC | 292 | 69(34-89) | CHT | 3 | 198/94 | Before therapy | 40.2(0.5-100.4) | 7 |
| **Roy Mano[7]** | Israel | 2014 | BC | 107 | 68(IQR:61-78) | TURBT | 2.41;2.43 | 68/39 | Before TURBT | 40(IQR:23-51) | 9 |
| **T Hermanns[8]** | Canada | 2014 | BC | 424 | 70.1(60.6-76.3) | RC | 3 | 216/208 | Before RC | 58.4(IQR:21.3-94.5) | 8 |
| **Boyd R. Viers[9]** | USA | 2014 | BC | 899 | 69(IQR:62-76) | RC | 2.7 | 569/343 | Within 3 months before RC | 10.9y(IQR:8.3-13.9) | 9 |
| **Haolun Luo[10]** | Taiwan | 2014 | UTUC | 234 | 67 | RNU | 3 | 94/140 | Within 1 week before RNU | 40.7±23.8 | 8 |
| **Hyun Hwan Sung[11]** | Korea | 2015 | UTUC | 410 | 64(IQR:55-72) | RNU | 2.5 | 146/264 | Before any manipulation | 40.2(33-66.1) | 8 |
| **Su Min Lee[12]** | UK | 2015 | BC | 226 | 75(IQR:65-81) | TURBT | 3.89 | 56/170 | Within 60 days before TURBT | NA | 6 |
| **Satoru Taguchi[13]** | Japan | 2015 | mUC | 185 | 68(IQR:62-74.5) | CHT | 3 | 93/92 | Within 1 week before CHT | 13.0(IQR:7-25.5) | 6 |
| **Myong Kim[14]** | Korea | 2015 | UTUC | 277 | 63.7（IQR:57.4-70.6） | RNU | 5 | 19/258 | Before RNU | NA | 8 |
| **Yen-Chen Cheng[15]** | Taiwan | 2015 | UTUC | 195 | 68±10.3 | RNU | 2.7 | NA | Within 2 weeks before RNU | 36 | 7 |
| **Cihat Ozcan[16]** | Turkey | 2015 | BC | 363 | 60.7（29-83） | RC | 2.5 | NA | Before RC; close to the date of surgery | 28(0-144) | 7 |
| **Minyong Kang[17]** | Korea | 2015 | BC | 385 | NA | RC | 2 | NA | Within 1 month before RC | 38 | |
| **Xin Song[18]** | China | 2016 | UTUC | 140 | 67(39-81) | RNU | 2.2 | 63/77 | Within 3 days before RNU | | 7 |

| | | | | | | | | | | | |
|---|---|---|---|---|---|---|---|---|---|---|---|
| **Yosuke Morizawa[19]** | Japan | 2016 | BC | 110 | 72(65-76) | RC | 2.6 | 55/55 | Before RC | 37.5(11-65) | 8 |
| **L.Spencer Krane[20]** | USA | 2013 | BC | 68 | 67.4±10.1 | RC | 2.5 | NA | Before RC | NA | 7 |
| **Bimal Bhindi[21]** | Canada | 2015 | BC | 418 | 70(IQR:61-76) | RC | 2.9 | 209/209 | 1 week before RC | 40(IQR14-72) | 7 |
| **Takashi Kawahara[22]** | Japan | 2016 | BC | 74 | 65 | RC | 2.38 | 23/51 | A few days before RC | median24.2 | 8 |
| **Mihai Dorin Vartolomei[23]** | austria | 2016 | UTUC | 2477 | 69(IQR:61-76) | RNU | 2.7 | 1428/1049 | Within 1 month before RNU | 40(IQR:20-76) | 9 |
| **Guiming Zhang[24]** | China | 2015 | BC | 124 | 65(30-78) | RC | 2.1 | 52/72 | Within 3 days before surgery | 50.8 | 8 |
| **JH Ku[25]** | Korea | 2015 | BC | 419 | 65.1(58.3-70.4) | RC | 5 | NA | Before RC | 37.7(IQR:20.5-70.7) | 7 |
| **Richard M Bambury[26]** | USA | 2015 | UC | 129 | 66(45-85) | CHT | 2.5 | NA | Before CHT | NA | 7 |
| **Koichiro Ogihara[27]** | Japan | 2016 | BC | 605 | 68(21-94) | TURBT | 2.2 | 296/309 | Before TURBT | 68.8（4.5-237） | 9 |
| **Oscar Buisan[28]** | Spain | 2016 | BC | 75 | NLR<2.5:64.6(45-78)<br>NLR>2.5:69.2(53-83) | NAC,RC | 2.5 | 34/41 | Within 3 months before treatment | NLR<2.5:25.4(3-97)<br>NLR>2.5:29.1(3-101) | 8 |
| **Vincenzo Favilla[29]** | Italy | 2016 | BC | 178 | 69.3(IQR:63.8-79.4) | TURBT | 3 | 60/118 | Before TURBT | 53(IQR:33-76) | 7 |
| **Shinji Ohtake[30]** | Japan | 2016 | BC | 23 | 63(46-74) | RC,CHT | 4.14 | 14/9 | A few days before chemotherapy | 11.5(2.3-29.8) | 6 |
| **Aurélie Mbeutcha[31],** | European | 2016 | BC | 1117 | 67(IQR58-74) | TURBT | 2.5 | 360/757 | More than 1 week before surgery | 64(IQR:26-100) | 8 |
| **Nozomu Kishimoto[32]** | Japan | 2016 | UTUC | 100 | 73(IQR：67-78) | RNU | 3.8 | 21/79 | Within 3 months before RNU | 34(IQR:4-191) | 6 |

NLR, Neutrophil-to-lymphocyte ratio; NOS, Newcastle-ottawa quality assessment scale; BC, bladder cancer; UTUC, upper tract urothelial carcinoma; RC, radical cystectomy; RNU, radical nephroureterectomy; CHT, chemotherapy; NAC, neoadjuvant chemotherapy; IQR, interquartile range.

**Supplementary Table 3 NLR and Prognosis correlation results of included studies**

| Study | OS Univariate | | | OS Multivariate | | | PFS Univariate | | | PFS Multivariate | | | CSS Univariate | | | CSS Multivariate | | |
|---|---|---|---|---|---|---|---|---|---|---|---|---|---|---|---|---|---|---|
| | **HR** | **LCI** | **UCI** | **HR** | **LCI** | **UCI** | **HR** | **LCI** | **UCI** | **HR** | **LCI** | **UCI** | **HR** | **LCI** | **UCI** | **HR** | **LCI** | **UCI** |
| **Tatsuo Gondo** | NA | NA | NA | NA | NA | NA | NA | NA | NA | NA | NA | NA | NA | NA | NA | 1.95 | 1.04 | 3.66 |
| **Takeshi Azuma** | NA | NA | NA | NA | NA | NA | 4.48 | 2.64 | 7.8 | 2.11 | 1.02 | 4.46 | 6.14 | 3.52 | 11.2 | 3.06 | 1.44 | 6.83 |
| **Orietta Dalpiaz** | 3.07 | 1.67 | 5.67 | 2.48 | 1.31 | 4.70 | NA | NA | NA | NA | NA | NA | 3.11 | 1.47 | 6.59 | 2.72 | 1.25 | 5.93 |
| **Nobuyuki Tanaka** | NA | NA | NA | NA | NA | NA | 1.75 | 1.3 | 2.36 | 1.38 | 1.02 | 1.87 | 2.07 | 1.45 | 2.94 | 1.47 | 1.03 | 2.11 |
| **Lorena Rossi** | 1.74 | 1.32 | 2.29 | NA | NA | NA | 1.51 | 1.16 | 1.96 | NA | NA | NA | NA | NA | NA | NA | NA | NA |
| **Roy Mano** | NA | NA | NA | NA | NA | NA | 3.74 | 1.43 | 9.79 | 3.52 | 1.33 | 9.33 | NA | NA | NA | NA | NA | NA |
| **T Hermanns** | 1.8 | 1.48 | 2.2 | 1.67 | 1.17 | 2.40 | 1.53 | 1.23 | 1.89 | 1.49 | 1.12 | 2 | 1.88 | 1.52 | 2.33 | 1.88 | 1.39 | 2.54 |
| **Boyd R. Viers** | 1.61 | 1.35 | 1.92 | NA | NA | NA | NA | NA | NA | NA | NA | NA | 1.47 | 1.18 | 1.83 | NA | NA | NA |
| **Hao-Lun Luo** | NA | NA | NA | NA | NA | NA | NA | NA | NA | 2.47 | 1.16 | 5.29 | NA | NA | NA | 6.38 | 1.18 | 23.31 |
| **Hyun Hwan Sung** | 1.83 | 1.36 | 2.47 | NA | NA | NA | 2.0 | 1.4 | 2.86 | 1.70 | 1.14 | 2.56 | 2.04 | 1.42 | 2.91 | NA | NA | NA |
| **Satoru Taguchi** | NA | NA | NA | 1.49 | 1.02 | 2.18 | NA | NA | NA | NA | NA | NA | NA | NA | NA | 1.48 | 1.01 | 2.17 |
| **Myong Kim** | NA | NA | NA | NA | NA | NA | 1.21 | 0.53 | 2.78 | NA | NA | NA | 1.18 | 0.51 | 2.72 | NA | NA | NA |
| **Yen-Chen Cheng** | 2.62 | 1.26 | 3.73 | 1.61 | 0.89 | 2.92 | NA | NA | NA | NA | NA | NA | 2.23 | 1.16 | 4.28 | 1.36 | 0.65 | 2.85 |
| **Cihat Ozcan** | NA | NA | NA | NA | NA | NA | NA | NA | NA | NA | NA | NA | 1.80 | 1.26 | 2.57 | 1.97 | 1.042 | 3.59 |
| **Minyong Kang** | NA | NA | NA | 1.13 | 1.04 | 1.22 | NA | NA | NA | NA | NA | NA | NA | NA | NA | 1.16 | 1.06 | 1.28 |
| **Xin Song** | NA | NA | NA | NA | NA | NA | NA | NA | NA | 3.82 | 1.49 | 9.76 | NA | NA | NA | NA | NA | NA |
| **Yosuke Morizawa** | 2.7 | 1.4 | 5.2 | 2.8 | 1.4 | 5.4 | 2.4 | 1.3 | 4.6 | 2.6 | 1.1 | 6 | 2.9 | 1.4 | 6.2 | 2.6 | 1.9 | 5.2 |
| **L.Spencer Krane** | 2.25 | 1.08 | 5.29 | 2.49 | 1.14 | 6.09 | NA | NA | NA | NA | NA | NA | NA | NA | NA | NA | NA | NA |
| **Bimal Bhindi** | 1.92 | 1.46 | 2.53 | NA | NA | NA | 1.85 | 1.34 | 2.55 | NA | NA | NA | 1.71 | 1.21 | 2.4 | NA | NA | NA |
| **Guiming Zhang** | 1.23 | 0.64 | 2.39 | NA | NA | NA | NA | NA | NA | NA | NA | NA | NA | NA | NA | NA | NA | NA |
| **JH Ku** | 2.33 | 1.41 | 3.83 | 2.29 | 1.35 | 3.87 | 2.27 | 1.27 | 4.08 | 2.02 | 1.09 | 3.77 | NA | NA | NA | NA | NA | NA |
| **Richard M** | 1.76 | 0.85 | 3.62 | NA | NA | NA | NA | NA | NA | NA | NA | NA | NA | NA | NA | NA | NA | NA |

| | | | | | | | | | | | | | | | | | | |
|---|---|---|---|---|---|---|---|---|---|---|---|---|---|---|---|---|---|---|
| **Bambury** | | | | | | | | | | | | | | | | | | |
| **Takashi Kawahara** | 4.84 | 1.54 | 15.2 | 4.62 | 1.16 | 18.3 | 2.0 | 0.84 | 4.80 | NA | NA | NA | NA | NA | NA | NA | NA | NA |
| **Mihai Dorin Vartolomei** | NA | NA | NA | NA | NA | NA | 1.3 | 1.09 | 1.56 | 1.05 | 0.87 | 1.26 | NA | NA | NA | NA | NA | NA |
| **Koichiro Ogihara** | NA | NA | NA | NA | NA | NA | NA | NA | NA | 2.37 | 1.17 | 4.78 | NA | NA | NA | NA | NA | NA |
| **Oscar Buisan** | 1.58 | 0.73 | 3.42 | NA | NA | NA | 1.84 | 0.85 | 3.96 | NA | NA | NA | 1.87 | 0.82 | 4.26 | NA | NA | NA |
| **Vincenzo Favilla** | NA | NA | NA | NA | NA | NA | NA | NA | NA | 5.35 | 0.39 | 73.7 | NA | NA | NA | NA | NA | NA |
| **Shinji Ohtake** | 0.8 | 0.1 | 6.6 | NA | NA | NA | 1.65 | 0.61 | 4.48 | NA | NA | NA | NA | NA | NA | NA | NA | NA |
| **Aurélie Mbeutcha,** | NA | NA | NA | NA | NA | NA | 1.76 | 1.2 | 2.58 | 1.72 | 1.16 | 2.54 | NA | NA | NA | NA | NA | NA |
| **Nozomu Kishimoto** | NA | NA | NA | NA | NA | NA | NA | NA | NA | NA | NA | NA | NA | NA | NA | NA | NA | NA |

NLR, Neutrophil-to-lymphocyte ratio; OS, overall survival; PFS, progression free survival; CSS, cancer specific survival; HR, hazard ratio; LCI, lower confidence interval; UCI, upper confidence interval

**Supplementary Table 4 NLR and clinical features correlation results of included studies**

| Study | LNI(H/L) | | LVI(H/L) | | CIS(H/L) | | PT stage(H/L) | | Tumor grade | | multifocality | | PM(H/L) | |
|---|---|---|---|---|---|---|---|---|---|---|---|---|---|---|
| | **PN0** | **pN+** | **N** | **P** | **N** | **P** | **pT1** | **pT2/3/4** | **G1/2** | **G3** | **N** | **P** | **N** | **P** |
| **Orietta Dalpiaz** | NA | NA | NA | NA | NA | NA | 49/30 | 68/24 | 57/35 | 60/19 | NA | NA | NA | NA |
| **Nobuyuki Tanaka** | 163/453 | 21/28 | 100/306 | 84/175 | 165/401 | 19/80 | 45/183 | 139/298 | 60/199 | 124/282 | 153/419 | 31/62 | NA | NA |
| **Mehmet Kaynar** | NA | NA | NA | NA | NA | NA | 43/149 | 32/67 | NA | NA | NA | NA | NA | NA |
| **Roy Mano** | NA | NA | NA | NA | 61/35 | 7/4 | NA | NA | 22/20 | 46/19 | 36/27 | 32/12 | NA | NA |
| **T Hermanns** | 155/153 | 53/63 | 139/142 | 77/66 | 131/103 | 85/105 | NA | NA | NA | NA | NA | NA | 200/191 | 16/17 |
| **Boyd R. Viers** | 398/342 | 100/49 | 372/308 | 132/88 | NA | NA | 198/194 | 305/199 | NA | NA | NA | NA | 481/382 | 19/7 |
| **Hao-Lun Luo** | NA | NA | 80/112 | 14/28 | 50/85 | 44/55 | 48/71 | 46/69 | 9/17 | 85/123 | 59/86 | 35/54 | NA | NA |
| **Su Min Lee** | NA | NA | NA | NA | NA | NA | 29/146 | 27/24 | 26/106 | 30/64 | 43/106 | 13/64 | NA | NA |
| **Xin Song** | NA | NA | 59/75 | 4/2 | NA | NA | NA | NA | 27/40 | 36/37 | NA | NA | NA | NA |
| **Yosuke Morizawa** | 42/46 | 13/9 | 37/42 | 18/13 | NA | NA | 11/24 | 44/31 | NA | NA | NA | NA | 45/52 | 10/3 |
| **Guiming Zhang** | NA | NA | NA | NA | 52/67 | 0/5 | 6/9 | 46/63 | 0/5 | 52/67 | NA | NA | NA | NA |
| **Takashi Kawahara** | 21/43 | 2/8 | NA | NA | NA | NA | NA | NA | NA | NA | NA | NA | 23/45 | 0/6 |
| **MD Vartolomei** | 1292/798 | 136/48 | 1072/703 | 356/143 | 1083/663 | 345/183 | 615/462 | 813/384 | 220/147 | 1208/699 | 1104/632 | 324/214 | NA | NA |
| **Koichiro Ogihara** | NA | NA | NA | NA | 27/37 | 269/272 | NA | NA | 189/178 | 107/131 | 150/144 | 146/165 | NA | NA |
| **Oscar Buisan** | 24/21 | 10/20 | 24/33 | 10/8 | NA | NA | 11/18 | 23/23 | NA | NA | NA | NA | 26/38 | 8/3 |
| **Vincenzo Favilla** | NA | NA | NA | NA | 24/98 | 36/20 | NA | NA | 36/90 | 24/28 | 28/90 | 32/28 | NA | NA |
| **Aurélie Mbeutcha,** | NA | NA | NA | NA | 342/709 | 18/48 | NA | NA | 187/342 | 173/315 | 240/478 | 120/279 | NA | NA |
| **Nozomu Kishimoto** | 17/75 | 4/4 | 14/58 | 7/21 | 17/59 | 4/19 | 10/43 | 11/36 | 11/40 | 10/39 | 19/68 | 2/11 | 20/75 | 1/4 |

NLR, Neutrophil-to-lymphocyte ratio; LNI, lymphnode involvement; LVI, lymphovascular invasion; CIS, carcinoma in situ

**Supplementary Table 5 Heterogeneity test and publication bias analyses among studies included**

| | OS | | | | PFS | | | | CSS | | | |
|---|---|---|---|---|---|---|---|---|---|---|---|---|
| | Pa | I2(%) | Pc | Pd | Pa | I2(%) | Pc | Pd | Pa | I2(%) | Pc | Pd |
| Overall | 0 | 71.6 | 0.329 | 0.414 | 0.021 | 44.2 | 0.502 | 0.597 | 0 | 67.2 | 0.583 | 0.908 |
| Geographic area | | | | | | | | | | | | |
| 1.Asian | 0 | 71 | 0.26 | 0.181 | 0.481 | 0 | 0.251 | 0.174 | 0 | 68.9 | 0.734 | 0.632 |
| 2.non-Asian | 0.863 | 0 | 1 | 0.711 | 0.02 | 60.1 | 1 | 0.331 | 0.01 | 66.7 | 0.251 | 0.798 |
| statistical methods | | | | | | | | | | | | |
| 1.univariate | 0.324 | 11.3 | 0.242 | 0.165 | 0.011 | 51.2 | 0.075 | 0.042 | 0.001 | 64.7 | 0.077 | 0.078 |
| 2.multivariate | 0 | 73.8 | 0.006 | 0 | 0.004 | 58.6 | 0 | 0 | 0 | 71.4 | 0.016 | 0.001 |
| Patient | | | | | | | | | | | | |
| 1.Localized | 0.605 | 0 | 0.06 | 0.004 | 0.009 | 50.3 | 0.945 | 0.822 | 0.005 | 55.2 | 0.876 | 0.89 |
| 2.metastatic | 0.822 | 0 | 1 | NA | 0.866 | 0 | NA | NA | NA | NA | NA | NA |
| sample size | | | | | | | | | | | | |
| 1.<230 | 0.53 | 0 | 1 | 0.838 | 0.865 | 0 | 0.089 | 0.009 | 0.407 | 2.4 | 0.221 | 0.234 |
| 2.>=230 | 0 | 85.3 | 0.764 | 0.499 | 0.021 | 44.2 | 0.466 | 0.828 | 0 | 71 | 1 | 0.877 |
| NLR standard | | | | | | | | | | | | |
| 1.<2.65 | 0.001 | 71.1 | 0.764 | 0.534 | 0.764 | 0 | 1 | 0.915 | 0 | 78.1 | 1 | 0.005 |
| 2.>2.65 | 0.791 | 0 | 0.573 | 0.52 | 0.045 | 47.7 | 1 | 0.928 | 0.028 | 51.9 | 0.371 | 0.051 |
| follow-up | | | | | | | | | | | | |
| 1.=<39 | 0.005 | 66.8 | 0.851 | 0.124 | 0.686 | 0 | 1 | 0.359 | 0.017 | 58.8 | 1 | 0.633 |
| 2.>39 | 0.73 | 0 | 0.707 | 0.289 | 0.007 | 58.7 | 0.118 | 0.34 | 0.001 | 72.1 | 0.133 | 0.149 |
| tumor type | | | | | | | | | | | | |
| 1.UTUC | 0.735 | 0 | 1 | 0.986 | 0.014 | 60.3 | 1 | 0.966 | 0.005 | 63.3 | 1 | 0.942 |
| 2.BC | 0 | 75.9 | 0.297 | 0.449 | 0.021 | 44.2 | 0.23 | 0.371 | 0 | 73.4 | 0.548 | 0.6 |

NLR, Neutrophil-to-lymphocyte ratio; OS, overall survival; PFS, progression free survival; CSS, cancer specific survival; BC, bladder cancer; UTUC, upper tract urothelial carcinoma

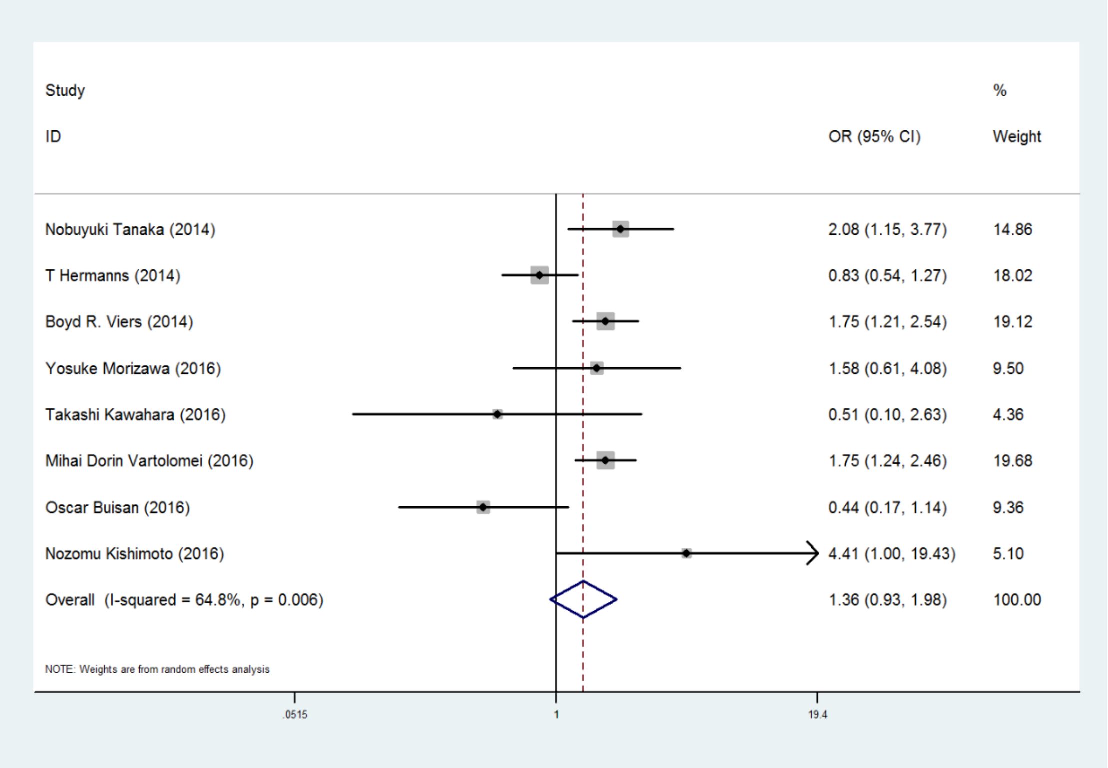


Supplementary Figure 1 Meta-analysis of correlation between NLR and LNI

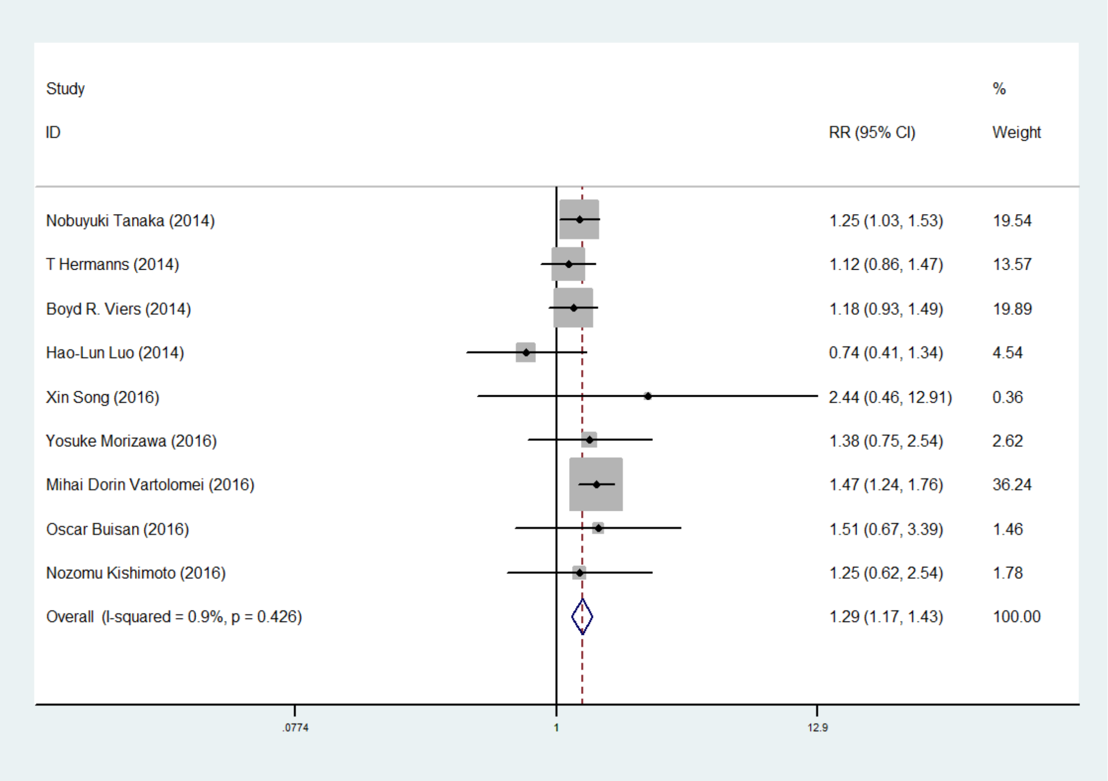


Supplementary Figure 2 Meta-analysis of correlation between NLR and LVI

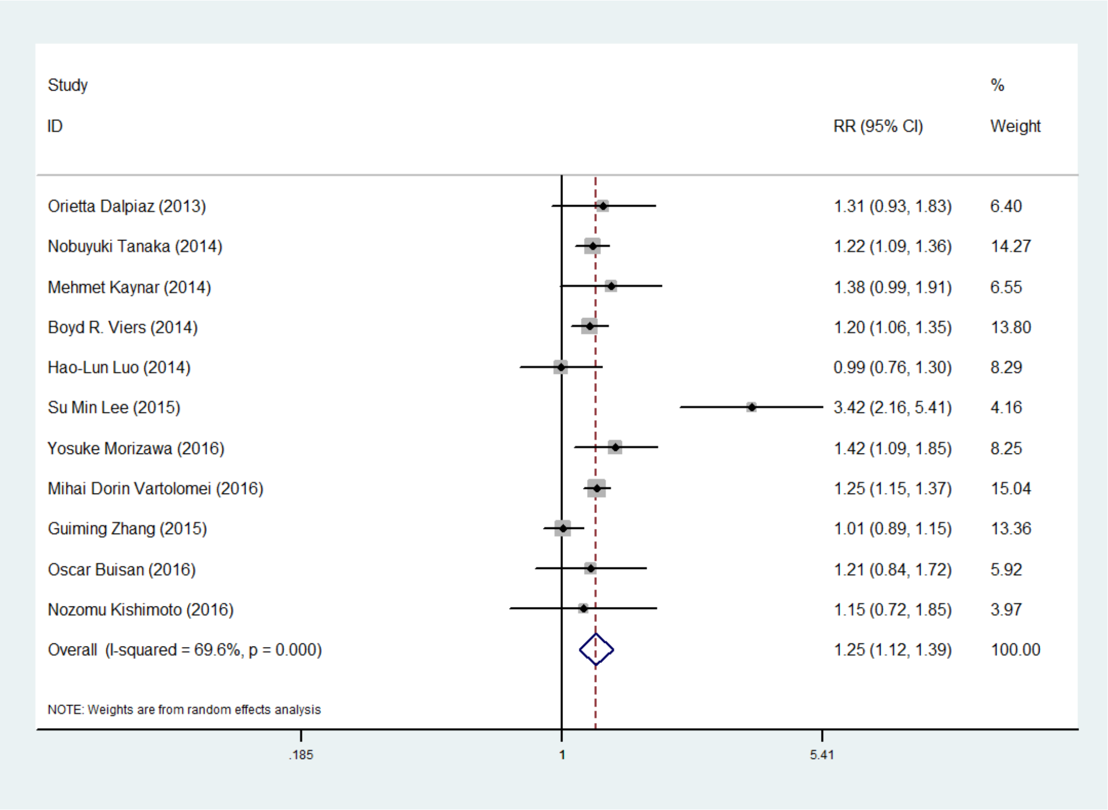


Supplementary Figure 3 Meta-analysis of correlation between NLR and T stage

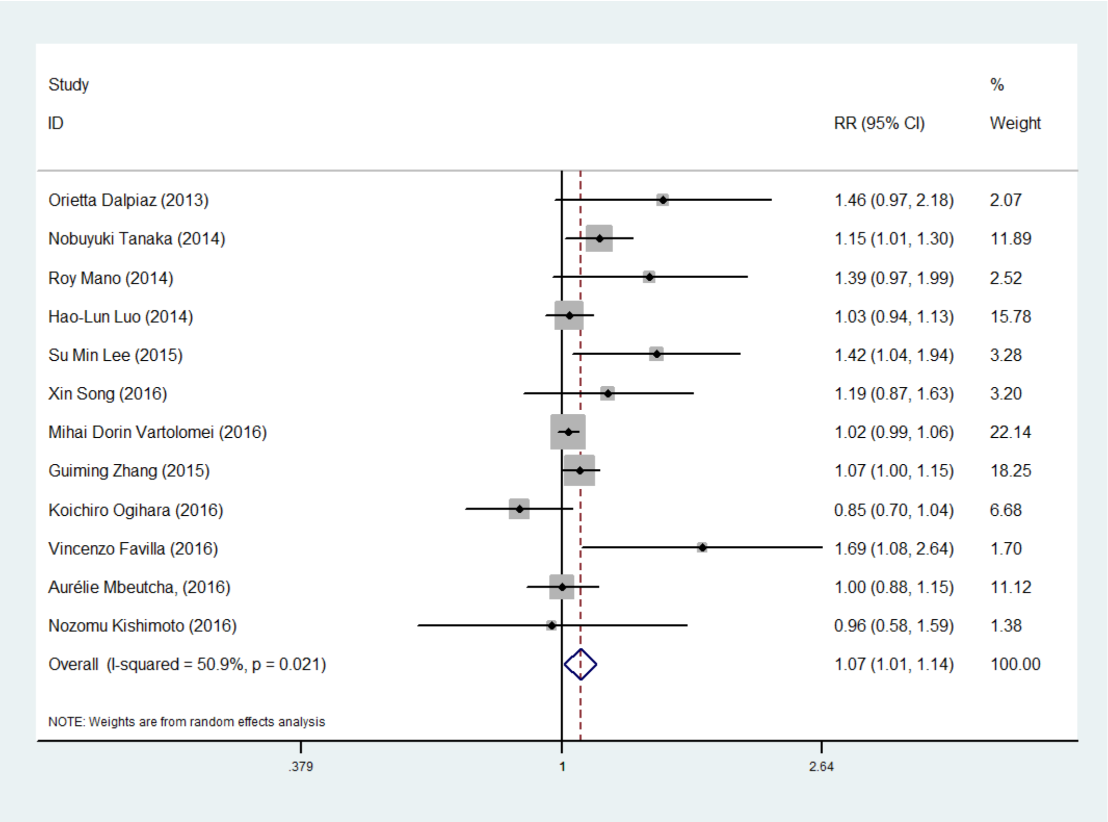


Supplementary Figure 4 Meta-analysis of correlation between NLR and tumor grade

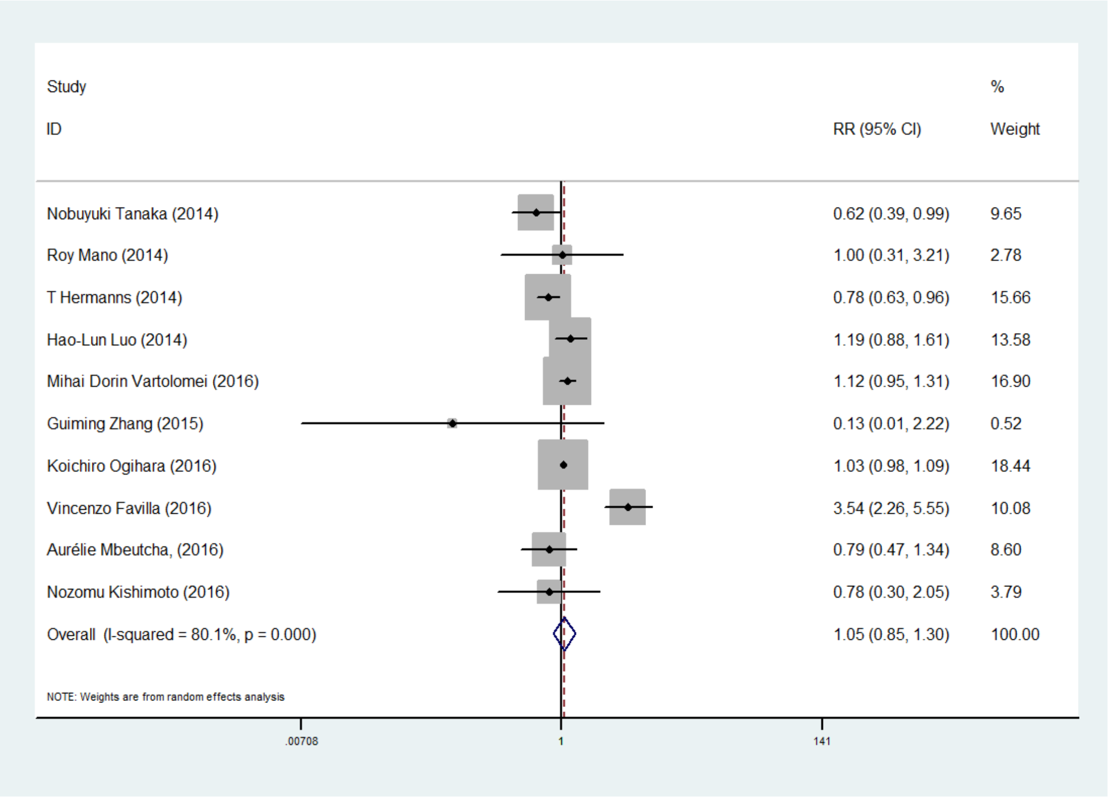

Supplementary Figure 5 Meta-analysis of correlation between NLR and CIS

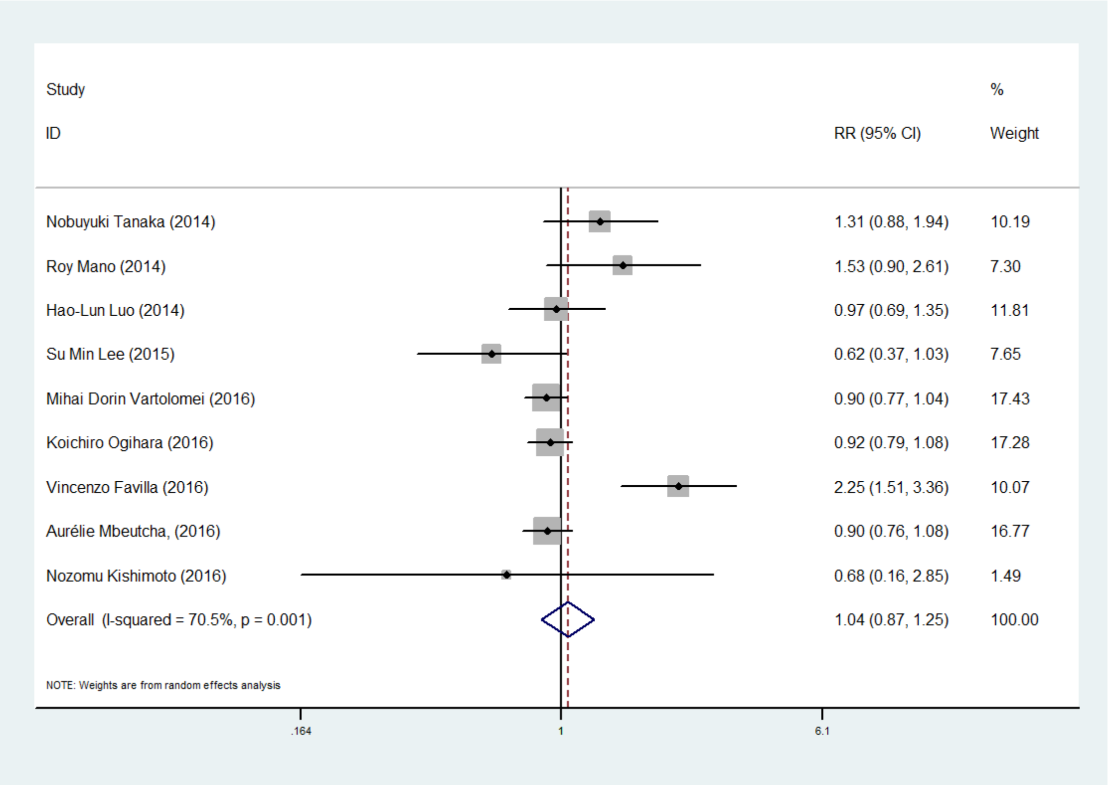


Supplementary Figure 6 Meta-analysis of correlation between NLR and multifocalty

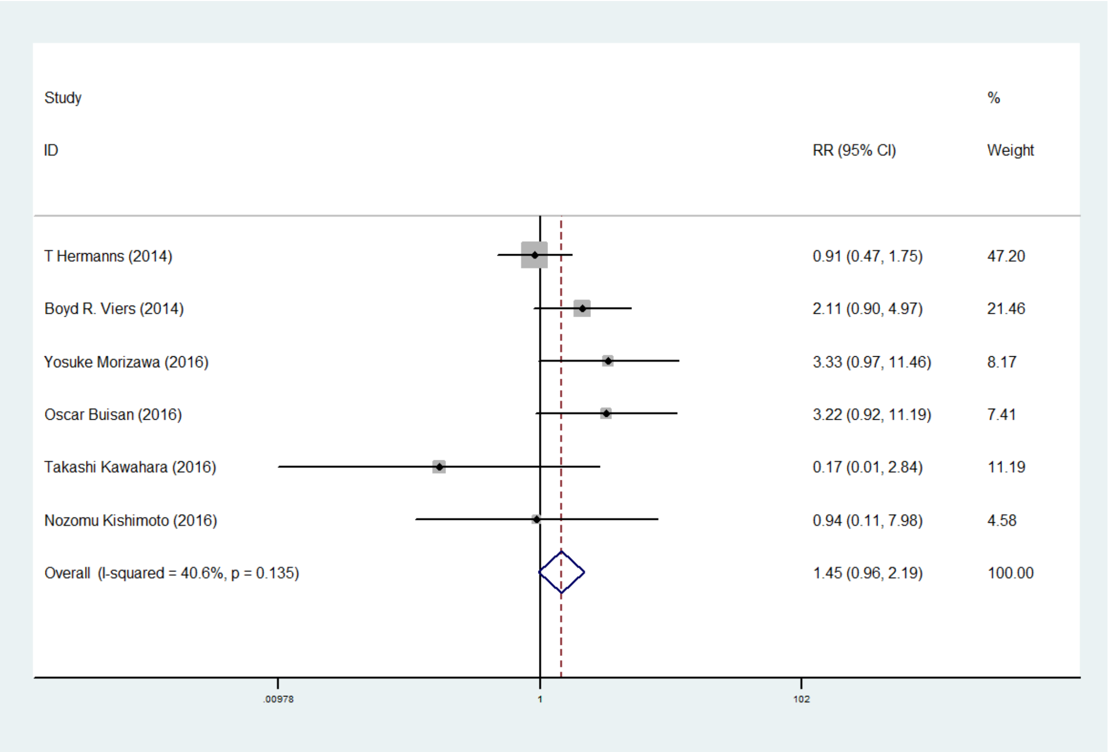

Supplementary Figure 7 Meta-analysis of correlation between NLR and positive margin

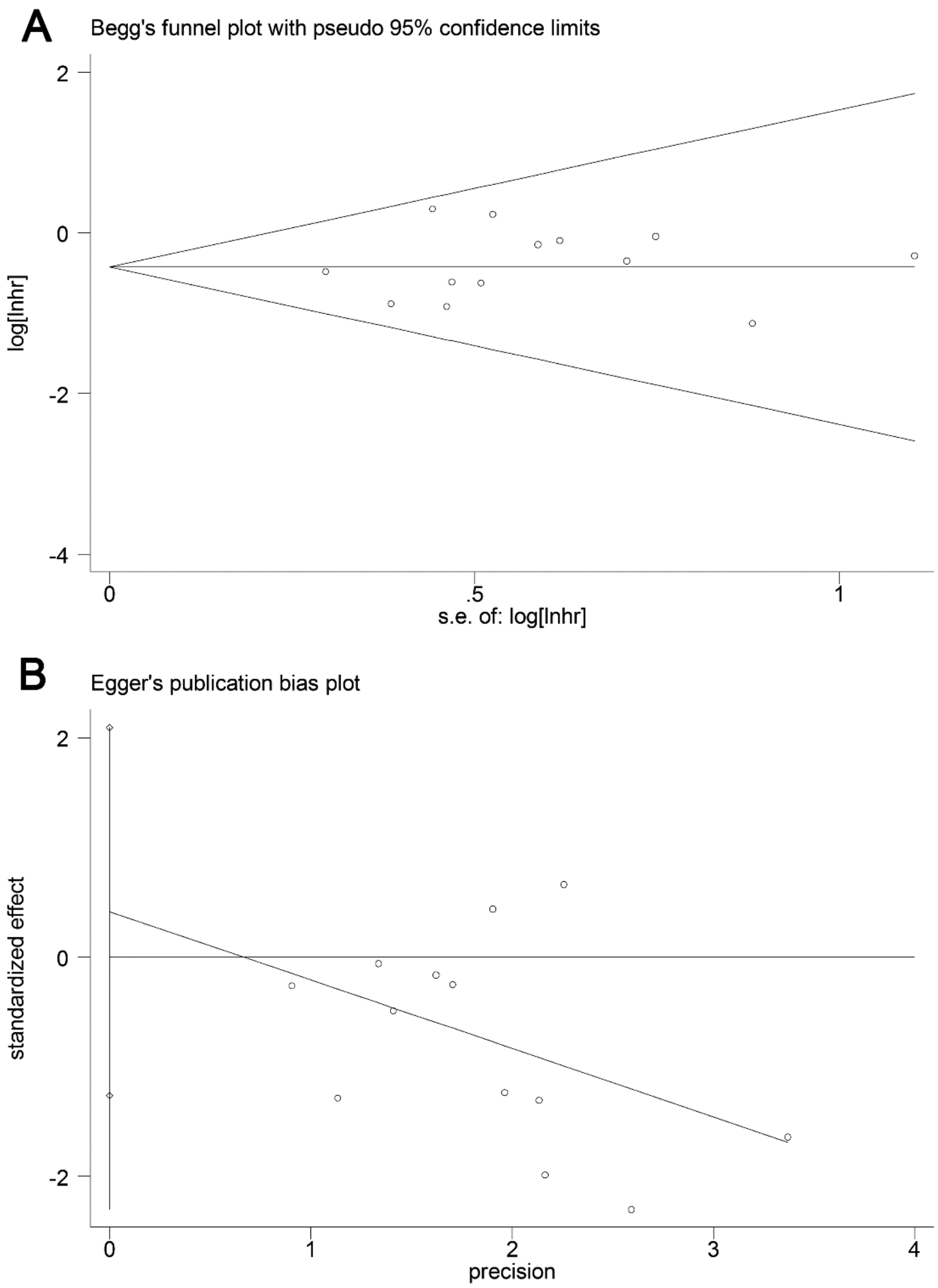


Supplementary Figure 8  Begg’s and Egger’s test for PFS

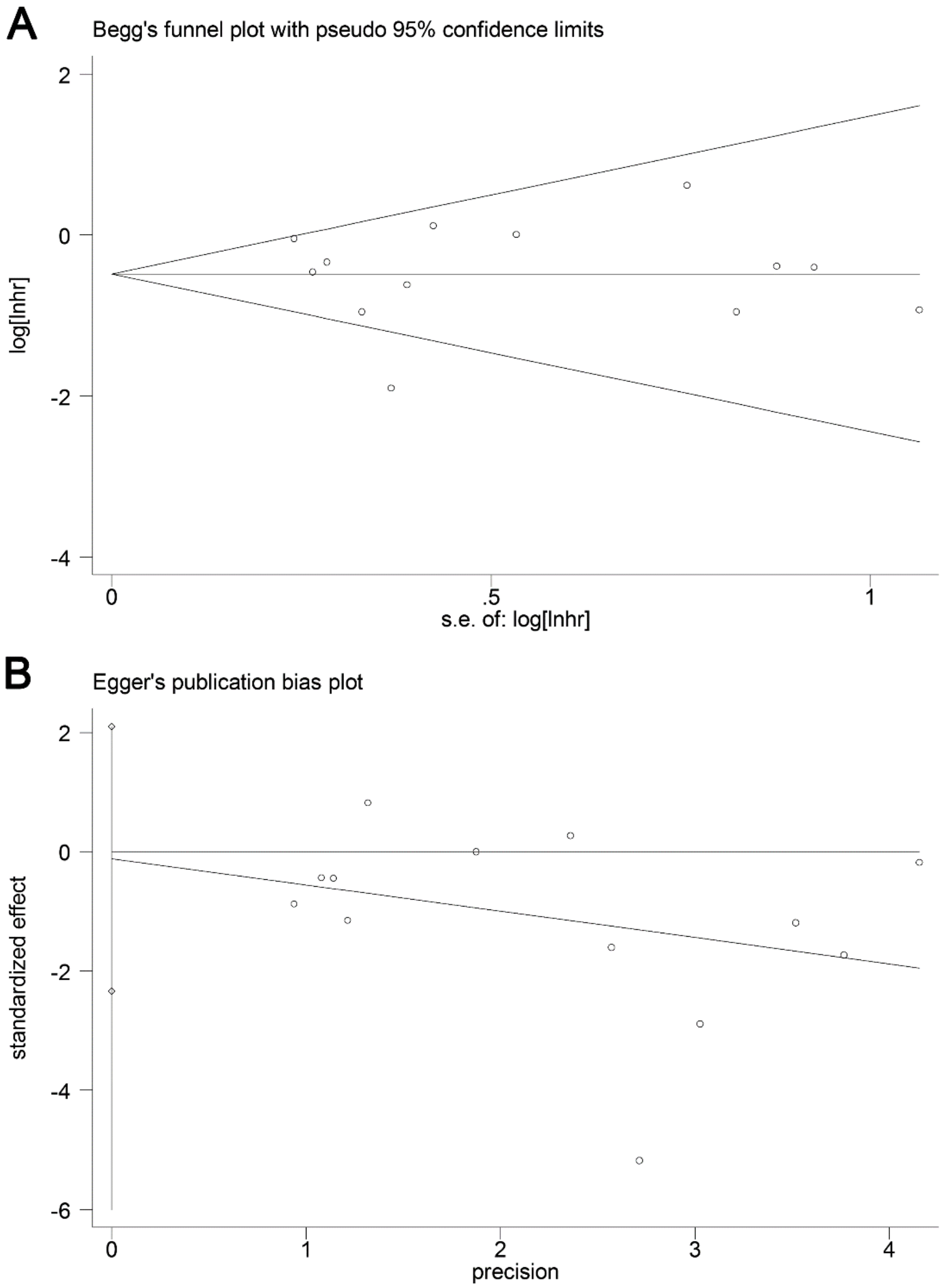


Supplementary Figure 9    Begg’s and Egger’s test for CSS

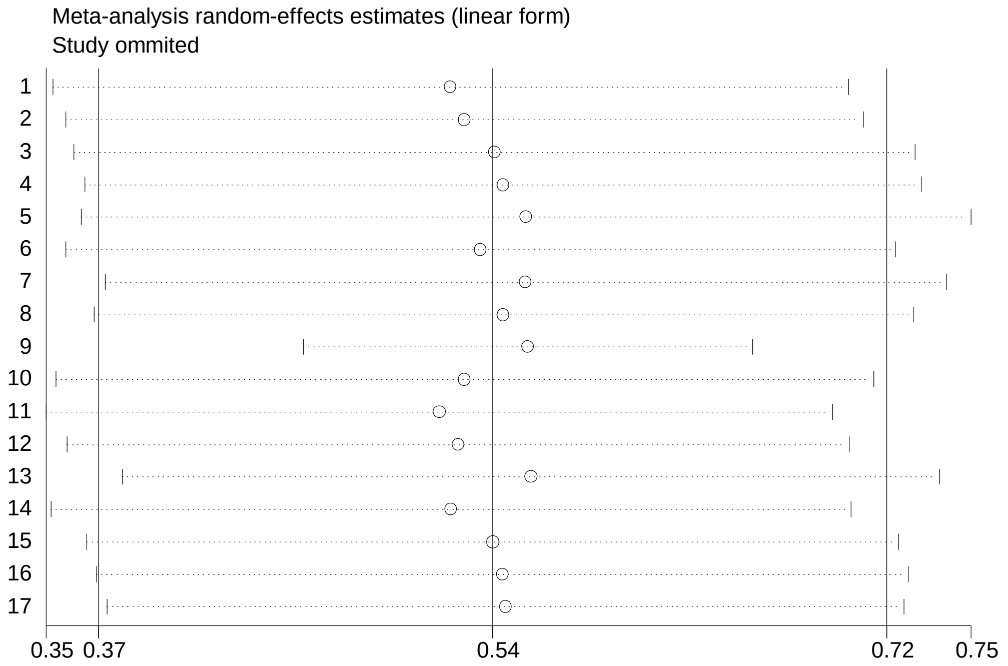


Supplementary Figure 10 Sensitivity analysis for OS

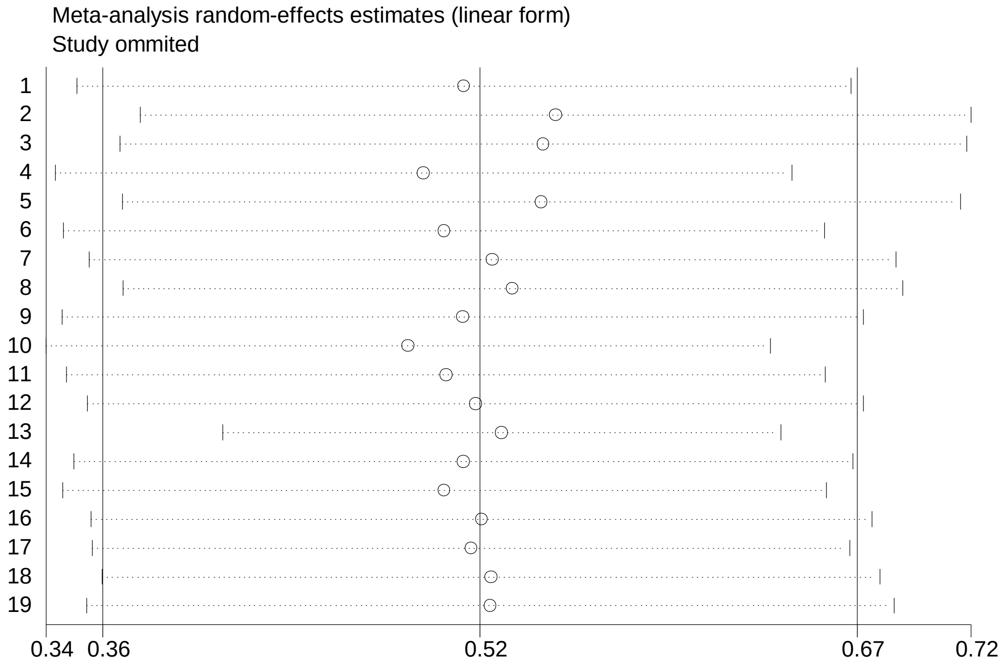


Supplementary Figure 11 Sensitivity analysis for PFS

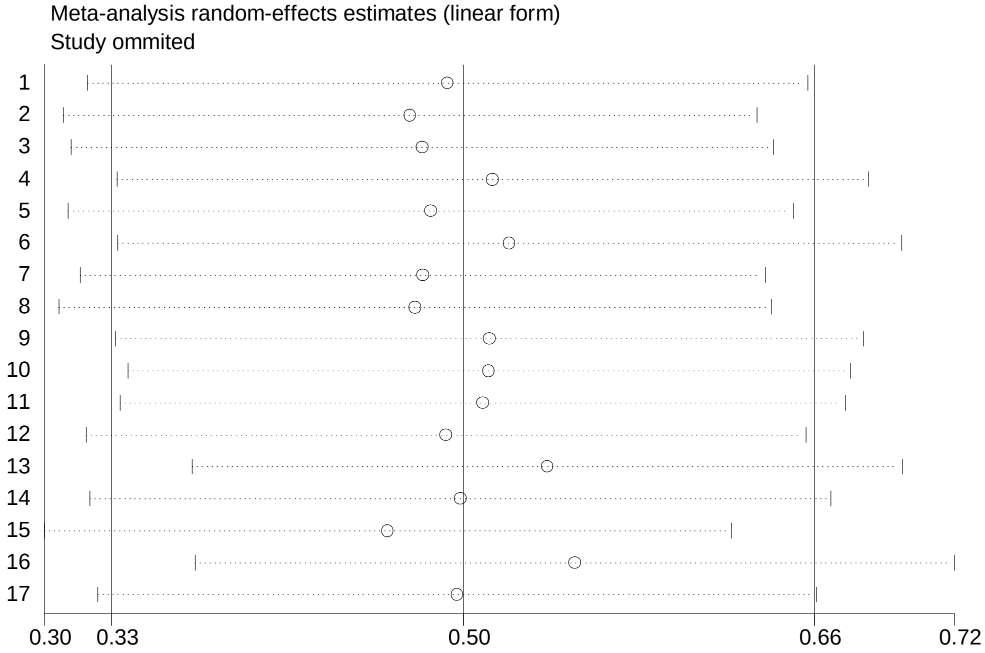


Supplementary Figure 12 Sensitivity analysis for CSS

## References


1. Gondo T, Nakashima J, Ohno Y, Choichiro O, Horiguchi Y, Namiki K, Yoshioka K, Ohori M, Hatano T and Tachibana M. Prognostic value of neutrophil-to-lymphocyte ratio and establishment of novel preoperative risk stratification model in bladder cancer patients treated with radical cystectomy. Urology. 2012; 79(5):1085-1091.
2. Azuma T, Matayoshi Y, Odani K, Sato Y, Sato Y, Nagase Y and Oshi M. Preoperative neutrophil-lymphocyte ratio as an independent prognostic marker for patients with upper urinary tract urothelial carcinoma. Clinical genitourinary cancer. 2013; 11(3):337-341.
3. Dalpiaz O, Ehrlich GC, Mannweiler S, Hernandez JM, Gerger A, Stojakovic T, Pummer K, Zigeuner R, Pichler M and Hutterer GC. Validation of pretreatment neutrophil-lymphocyte ratio as a prognostic factor in a European cohort of patients with upper tract urothelial carcinoma. BJU international. 2014; 114(3):334-339.
4. Tanaka N, Kikuchi E, Kanao K, Matsumoto K, Shirotake S, Miyazaki Y, Kobayashi H, Kaneko G, Hagiwara M, Ide H, Obata J, Hoshino K, Hayakawa N, Kosaka T, Hara S, Oyama M, et al. A Multi-Institutional Validation of the Prognostic Value of the Neutrophil-to-Lymphocyte Ratio for Upper Tract Urothelial Carcinoma Treated with Radical Nephroureterectomy. Annals of surgical oncology. 2014; 21(12):4041-4048.

5. Kaynar M, Yildirim ME, Badem H, Cavis M, Tekinarslan E, Istanbulluoglu MO, Karatas OF and Cimentepe E. Bladder cancer invasion predictability based on preoperative neutrophil-lymphocyte ratio. Tumour biology : the journal of the International Society for Oncodevelopmental Biology and Medicine. 2014; 35(7):6601-6605.
6. Rossi L, Santoni M, Crabb SJ, Scarpi E, Burattini L, Chau C, Bianchi E, Savini A, Burgio SL, Conti A, Conteduca V, Cascinu S and De Giorgi U. High Neutrophil-to-lymphocyte Ratio Persistent During First-line Chemotherapy Predicts Poor Clinical Outcome in Patients with Advanced Urothelial Cancer. Annals of surgical oncology. 2015; 22(4):1377-1384.
7. Mano R, Baniel J, Shoshany O, Margel D, Bar-On T, Nativ O, Rubinstein J and Halachmi S. Neutrophil-to-lymphocyte ratio predicts progression and recurrence of non-muscle-invasive bladder cancer. Urologic Oncology: Seminars and Original Investigations. 2015; 33(2):67e61-67e67.
8. Hermanns T, Bhindi B, Wei Y, Yu J, Noon AP, Richard PO, Bhatt JR, Almatar A, Jewett MA, Fleshner NE, Zlotta AR, Templeton AJ and Kulkarni GS. Pre-treatment neutrophil-to-lymphocyte ratio as predictor of adverse outcomes in patients undergoing radical cystectomy for urothelial carcinoma of the bladder. British journal of cancer. 2014; 111(3):444-451.
9. Viers BR, Boorjian SA, Frank I, Tarrell RF, Thapa P, Karnes RJ, Thompson RH and Tollefson MK. Pretreatment neutrophil-to-lymphocyte ratio is associated with advanced pathologic tumor stage and increased cancer-specific mortality among patients with urothelial carcinoma of the bladder undergoing radical cystectomy. European urology. 2014; 66(6):1157-1164.
10. Luo HL, Chen YT, Chuang YC, Cheng YT, Lee WC, Kang CH and Chiang PH. Subclassification of upper urinary tract urothelial carcinoma by the neutrophil-to-lymphocyte ratio (NLR) improves prediction of oncological outcome. BJU international. 2014; 113(5 B):E144-E149.
11. Sung HH, Jeon HG, Jeong BC, Seo SI, Jeon SS, Choi HY and Lee HM. Clinical significance of prognosis using the neutrophil-lymphocyte ratio and erythrocyte sedimentation rate in patients undergoing radical nephroureterectomy for upper urinary tract urothelial carcinoma. BJU international. 2015; 115(4):587-594.
12. Lee SM, Russell A and Hellawell G. Predictive value of pretreatment inflammation-based prognostic scores (Neutrophil-to-lymphocyte ratio, platelet-to-lymphocyte ratio, and lymphocyte-to-monocyte ratio) for invasive bladder carcinoma. Korean journal of urology. 2015; 56(11):749-755.
13. Taguchi S, Nakagawa T, Matsumoto A, Nagase Y, Kawai T, Tanaka Y, Yoshida K, Yamamoto S, Enomoto Y, Nose Y, Sato T, Ishikawa A, Uemura Y, Fujimura T, Fukuhara H, Kume H, et al. Pretreatment neutrophil-to-lymphocyte ratio as an independent predictor of survival in patients with metastatic urothelial carcinoma: A multi-institutional study. International Journal of Urology. 2015; 22(7):638-643.

14. Kim M, Moon KC, Choi WS, Jeong CW, Kwak C, Kim HH and Ku JH. Prognostic value of systemic inflammatory responses in patients with upper urinary tract urothelial carcinoma. World journal of urology. 2015; 33(10):1439-1457.
15. Cheng YC, Huang CN, Wu WJ, Li CC, Ke HL, Li WM, Tu HP, Li CF, Chang LL and Yeh HC. The Prognostic Significance of Inflammation-Associated Blood Cell Markers in Patients with Upper Tract Urothelial Carcinoma. Annals of surgical oncology. 2016; 23(1):343-351.
16. Ozcan C, Telli O, Ozturk E, Suer E, Gokce MI, Gulpinar O, Oztuna D, Baltaci S and Gogus C. The prognostic significance of preoperative leukocytosis and neutrophil-to-lymphocyte ratio in patients who underwent radical cystectomy for bladder cancer. Canadian Urological Association journal = Journal de l'Association des urologues du Canada. 2015; 9(11-12):E789-794.
17. Kang M, Jeong CW, Kwak C, Kim HH and Ku JH. The Prognostic Significance of the Early Postoperative Neutrophil-to-Lymphocyte Ratio in Patients with Urothelial Carcinoma of the Bladder Undergoing Radical Cystectomy. Annals of surgical oncology. 2016; 23(1):335-342.
18. Song X, Zhang GM, Ma XC, Luo L, Li B, Chai DY and Sun LJ. Comparison of preoperative neutrophil-lymphocyte, lymphocyte-monocyte, and platelet-lymphocyte ratios in patients with upper urinary tract urothelial carcinoma undergoing radical nephroureterectomy. OncoTargets and therapy. 2016; 9:1399-1407.
19. Morizawa Y, Miyake M, Shimada K, Hori S, Tatsumi Y, Nakai Y, Anai S, Tanaka N, Konishi N and Fujimoto K. Neutrophil-to-lymphocyte ratio as a detection marker of tumor recurrence in patients with muscle-invasive bladder cancer after radical cystectomy. Urologic oncology. 2016; 34(6):257.e211-257.
20. Krane LS, Richards KA, Kader AK, Davis R, Balaji KC and Hemal AK. Preoperative neutrophil/lymphocyte ratio predicts overall survival and extravesical disease in patients undergoing radical cystectomy. Journal of Endourology. 2013; 27(8):1046-1050.
21. Bhindi B, Hermanns T, Wei Y, Yu J, Richard PO, Wettstein MS, Templeton A, Li K, Sridhar SS, Jewett MA, Fleshner NE, Zlotta AR and Kulkarni GS. Identification of the best complete blood count-based predictors for bladder cancer outcomes in patients undergoing radical cystectomy. British journal of cancer. 2016; 114(2):207-212.
22. Kawahara T, Furuya K, Nakamura M, Sakamaki K, Osaka K, Ito H, Ito Y, Izumi K, Ohtake S, Miyoshi Y, Makiyama K, Nakaigawa N, Yamanaka T, Miyamoto H, Yao M and Uemura H. Neutrophil-to-lymphocyte ratio is a prognostic marker in bladder cancer patients after radical cystectomy. BMC cancer. 2016; 16(1):185.
23. Vartolomei MD, Mathieu R, Margulis V, Karam JA, Roupret M, Lucca I, Mbeutcha A, Seitz C, Karakiewicz PI, Fajkovic H, Wood CG, Weizer AZ, Raman JD, Rioux-Leclercq N, Haitel A, Bensalah K, et al. Promising role of preoperative neutrophil-to-lymphocyte ratio in patients treated with radical nephroureterectomy. World journal of urology. 2016.

24. Zhang GM, Zhu Y, Luo L, Wan FN, Zhu YP, Sun LJ and Ye DW. Preoperative lymphocyte-monocyte and platelet-lymphocyte ratios as predictors of overall survival in patients with bladder cancer undergoing radical cystectomy. Tumour biology : the journal of the International Society for Oncodevelopmental Biology and Medicine. 2015; 36(11):8537-8543.
25. Ku JH, Kang M, Kim HS, Jeong CW, Kwak C and Kim HH. The prognostic value of pretreatment of systemic inflammatory responses in patients with urothelial carcinoma undergoing radical cystectomy. British journal of cancer. 2015; 112(3):461-467.
26. Bambury RM, Benjamin DJ, Chaim JL, Zabor EC, Sullivan J, Garcia-Grossman IR, Regazzi AM, Ostrovnaya I, Apollo A, Xiao H, Voss MH, Iyer G, Bajorin DF and Rosenberg JE. The safety and efficacy of single-agent pemetrexed in platinum-resistant advanced urothelial carcinoma: a large single-institution experience. The oncologist. 2015; 20(5):508-515.
27. Ogihara K, Kikuchi E, Yuge K, Yanai Y, Matsumoto K, Miyajima A, Asakura H and Oya M. The Preoperative Neutrophil-to-lymphocyte Ratio is a Novel Biomarker for Predicting Worse Clinical Outcomes in Non-muscle Invasive Bladder Cancer Patients with a Previous History of Smoking. Annals of surgical oncology. 2016.
28. Buisan O, Orsola A, Areal J, Font A, Oliveira M, Martinez R and Ibarz L. Low Pretreatment Neutrophil-to-Lymphocyte Ratio Predicts for Good Outcomes in Patients Receiving Neoadjuvant Chemotherapy Before Radical Cystectomy for Muscle Invasive Bladder Cancer. Clinical genitourinary cancer. 2016.
29. Favilla V, Castelli T, Urzi D, Reale G, Privitera S, Salici A, Russo GI, Cimino S and Morgia G. Neutrophil to lymphocyte ratio, a biomarker in non-muscle invasive bladder cancer: a single-institutional longitudinal study. International braz j urol : official journal of the Brazilian Society of Urology. 2016; 42(4):685-693.
30. Ohtake S, Kawahara T, Kasahara R, Ito H, Osaka K, Hattori Y, Teranishi JI, Makiyama K, Mizuno N, Umemoto S, Miyoshi Y, Nakaigawa N, Miyamoto H, Yao M and Uemura H. Pretreatment Neutrophil-to-Lymphocyte Ratio Can Predict the Prognosis in Bladder Cancer Patients Who Receive Gemcitabine and Nedaplatin Therapy. BioMed research international. 2016; 2016:9846823.
31. Mbeutcha A, Shariat SF, Rieken M, Rink M, Xylinas E, Seitz C, Lucca I, Mathieu R, Roupret M, Briganti A, Karakiewicz PI and Klatte T. Prognostic significance of markers of systemic inflammatory response in patients with non-muscle-invasive bladder cancer. Urologic oncology. 2016; 34(11):483 e417-483 e424.
32. Kishimoto N, Takao T, Kuribayashi S, Yamamichi G, Nakano K, Kawamura M, Tsutahara K, Tanigawa G and Yamaguchi S. The neutrophil-to-lymphocyte ratio as a predictor of intravesical recurrence in patients with upper urinary tract urothelial carcinoma treated with radical nephroureterectomy. International journal of clinical oncology. 2016.
